# Time-varying land subsidence detected by radar altimetry: California, Taiwan and north China

**DOI:** 10.1038/srep28160

**Published:** 2016-06-21

**Authors:** Cheinway Hwang, Yuande Yang, Ricky Kao, Jiancheng Han, C. K. Shum, Devin L. Galloway, Michelle Sneed, Wei-Chia Hung, Yung-Sheng Cheng, Fei Li

**Affiliations:** 1 Department of Civil Engineering, National Chiao Tung University, 1001 Ta Hsueh Road, Hsinchu, Taiwan; 2 Chinese Antarctic Center of Surveying and Mapping, Wuhan University, 129 Luoyu Road, Wuhan 430079, China; 3 State Key Laboratory of Geodesy and Earth Dynamics, Institute of Geodesy & Geophysics, Chinese Academy of Sciences, Wuhan 43077, China; 4 Division of Geodetic Science, School of Earth Sciences, the Ohio State University, Columbus, OH 43210, USA; 5 U.S. Geological Survey, 5957 Lakeside Blvd, Indianapolis, IN 46278, USA; 6 U.S. Geological Survey, 6000 J Street, Placer Hall, Sacramento, CA 95819, USA; 7 Green Environmental Engineering Consultant Co. LTD, Hsinchu, Taiwan

## Abstract

Contemporary applications of radar altimetry include sea-level rise, ocean circulation, marine gravity, and icesheet elevation change. Unlike InSAR and GNSS, which are widely used to map surface deformation, altimetry is neither reliant on highly temporally-correlated ground features nor as limited by the available spatial coverage, and can provide long-term temporal subsidence monitoring capability. Here we use multi-mission radar altimetry with an approximately 23 year data-span to quantify land subsidence in cropland areas. Subsidence rates from TOPEX/POSEIDON, JASON-1, ENVISAT, and JASON-2 during 1992–2015 show time-varying trends with respect to displacement over time in California’s San Joaquin Valley and central Taiwan, possibly related to changes in land use, climatic conditions (drought) and regulatory measures affecting groundwater use. Near Hanford, California, subsidence rates reach 18 cm yr^−1^ with a cumulative subsidence of 206 cm, which potentially could adversely affect operations of the planned California High-Speed Rail. The maximum subsidence rate in central Taiwan is 8 cm yr^−1^. Radar altimetry also reveals time-varying subsidence in the North China Plain consistent with the declines of groundwater storage and existing water infrastructure detected by the Gravity Recovery And Climate Experiment (GRACE) satellites, with rates reaching 20 cm yr^−1^ and cumulative subsidence as much as 155 cm.

Land subsidence is caused by natural and/or anthropogenic processes including subsurface fluid extraction, underground mining, drainage of organic soils, sediment compaction/load in coastal regions, and permafrost degradation^1^,^2^. Globally, in regions with irrigated agriculture and rapid population growth, groundwater extraction typically is the principal cause of subsidence^1^,^3^,^4^. Subsidence is a hazard that increases flood risk, causes damages to man-made structures and cultural heritages in low-lying regions, exacerbates sea level rise in coastal regions, and results in significant socio-economic distress^1^,^5^. Standard tools for monitoring subsidence are precision leveling, Global Navigation Satellite System (GNSS), interferometric synthetic aperture radar (InSAR) and borehole extensometers^1^,^4^,^6^. Satellite gravimetry from the Gravity Recovery And Climate Experiment (GRACE^7^) can deduce groundwater mass changes at a coarse spatial resolution (350 km or greater). Currently, a large percentage of lands including croplands is threatened by subsidence, erosion and desertification which are affecting global food safety^8^. By 2050, an estimated two billion more people will need to be fed, increasing demand on agricultural land use for improved rates of food production^9^. For irrigated croplands dependent solely on groundwater or on the conjunctive use of surface water and groundwater, high rates of groundwater extraction can lead to unsustainable cropland practices owing to groundwater depletion. These practices can result in undesirable effects including irreversible aquifer-system compaction and land subsidence in susceptible aquifer systems, which can be exacerbated during droughts^1^,^2^,^10^.

This study explores the use of satellite radar altimetry for improved monitoring of subsidence in three irrigated cropland areas: California’s San Joaquin Valley (SJV), central Taiwan (CT), and the North China Plain (NCP). The three croplands areas are affected by historical and ongoing subsidence^3^,^11^,^12^,^13^,^14^,^15^,^16^, where groundwater extraction is the leading cause of subsidence^2^,^6^,^12^,^16^, and recent droughts have exacerbated groundwater depletion and its consequences in the SJV and the NCP^13^,^17^. Subsidence poses risks to existing high speed railways in CT, the NCP, and one under construction in the SJV^4^. Subsidence patterns and rates in much of the NCP, SJV and CT have been extensively studied using precision leveling, extensometry, InSAR, and GNSS^4^,^6^,^14^,^15^,^18^.

A synthesis of vertical displacement rates (VDRs) measured at 1,499 GNSS stations (Table S1 and Fig. S1, Sec. S1, Supplementary Information [SI]) between 1993 and 2015 across the Central Valley (including the SJV and the southern portion of the Sacramento Valley), California, show that the distribution of the GNSS sites within the valley are too sparse by themselves to map areal subsidence in adequate spatial detail (Fig. 1). Three coalescing subsidence bowls are evident: a southern bowl south of Bakersfield corresponding to the Arvin-Maricopa historical subsidence bowl^19^, a central bowl between Bakersfield and Fresno roughly centered on the Tulare-Hanford area and roughly corresponding to the Tulare-Wasco historical subsidence bowl^19^, and a northern bowl north of Fresno roughly centered on the Madera-Mendota area near the Delta-Mendota Canal^15^, which is located along the western edge of the San Joaquin Valley (Fig. 1). In general, the GNSS-derived subsidence rates are relatively large in the Tulare-Hanford area (maximum: 9.8 cm yr^−1^), and the rates decay southward and northward in the SJV to near zero. In the mountainous regions surrounding the Central Valley, the VDRs are largely positive, suggesting uplifts possibly caused by tectonics and reduced mass loading due to the current drought in California^20^. The mean subsidence rate in the central subsidence bowl (from Bakersfield to Fresno) is 2.86 cm yr^−1^, compared with 0.62 cm yr^−1^ in the northern subsidence bowl and smaller mean rates in the Sacramento Valley. Note that a negative vertical displacement rate determined in this paper (in all relevant figures and tables) corresponds to a positive subsidence rate. To emphasize land subsidence in this paper, we replace negative displacement rates by subsidence rates in all descriptions.

Mean subsidence rates during 2002–14 in CT derived from annual precision leveling ranged from zero to about 8 cm yr^−1^ (Fig. 2a, Sec. S2, SI). Despite its high accuracy, precision leveling can be costly^4^ and generally is not repeated frequently enough to resolve inter-annual and annual signals. About 310,000 pumping wells in Changhua and Yunlin Counties pump groundwater to meet agricultural and industrial water demands (Fig. 2b). Two major coalescing subsidence bowls are evident—on either side of the Zhuoshui River, and subsidence rates as much as 7 cm yr^−1^ threaten the operation of the Taiwan High Speed Rail^4^ (THSR; Fig. 2a) and sustainable development in the region. For example, the prevalence of fine-grained sediments here makes this area more susceptible to compaction. Though pumping-well distribution is even, subsidence rates are not, suggesting a significant spatial variation of hydrogeological properties^6^.

The NCP has a population of 437 million and an area of 300,000 km^2^, and is one of many regions in China with critical subsidence problems^16^,^21^. In the NCP, groundwater provides more than 60% of fresh water supplies^22^ and is the main water source for agricultural irrigation^23^. Near Tianjin, the mean subsidence rates range from 0.80 to 5.60 cm yr^−1^, with a maximum rate of 16 cm yr^−1^ and cumulative subsidence of 3.90 m during 1965–85^18^,^24^. The GRACE satellite gravimetry data have detected mass losses that are attributed to severe groundwater storage declines in the SJV and NCP^13^,^22^.

In this paper, we explore satellite altimetry as a new remote-sensing subsidence-mapping method and demonstrate its utility in cropland areas where the differential and persistent scatterer (PS)-based InSAR methods are limited by sparse temporally-coherent, stable radar reflectors^3^; however, improvements in identifying PS scatterers over agricultural regions have been reported^25^,^26^. We also demonstrate the temporal and along-track spatial detail of the radar altimetry method compared to GNSS (Fig. S1) and leveling (Fig. S2) which can be cost prohibitive to map subsidence in similar temporal and spatial detail. Satellite altimetry has been principally used to derive marine gravity/bathymetry, and to study ocean circulations, sea level rise, ice sheet elevation and water level changes^27^,^28^,^29^. Because only a few studies have used satellite altimetry to measure solid earth deformation and a standard numerical procedure to measure vertical displacement has not yet been established, there are concerns about the accuracies of altimeter-derived rates^30^,^31^. However, this study shows that, accurate VDRs at the sub-cm yr^−1^ level can be achieved using satellite altimetry by using more than two decades of near-continuous land surface elevation measurements from the improved coverage offered from multi-mission altimeter data with 10-day or monthly sampling: TOPEX/POSEIDON (TP, 1992–2002), JASON-1 (J1, 2002–09), JASON-2 (J2, 2008–present), and ENVISAT (EN, 2002–10). TP, J1 and J2 have the same repeated ground tracks.

## Results

**Figure 1:**
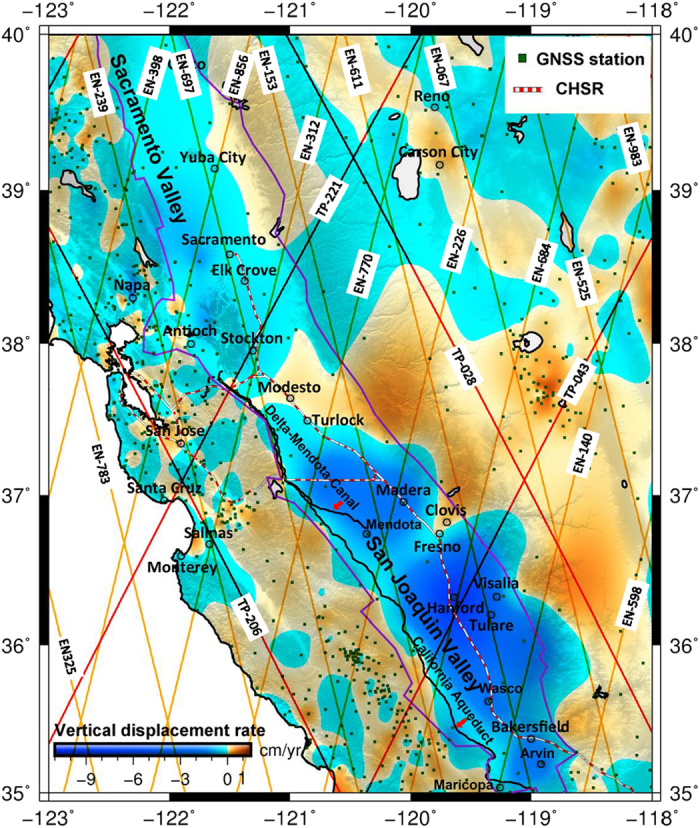
GNSS (green points)-derived vertical displacement rates (negative of subsidence rates) in the Central Valley (includes the San Joaquin and southern portion of the Sacramento Valleys inside the boundary in purple), California, (Sec. S1, SI). The planned California High-Speed Rail (CHSR) is red alternating with white line. GMT^36^ V5.1.3 is used to plot Fig. 1 (http://gmt.soest.hawaii.edu/). Overlapped are TOPEX/POSEIDON (red line) and ENVISAT (orange line) satellite ground tracks (labeled as satellite abbreviation-pass number), where vertical displacement rates at a 1-km interval are derived from the two altimeters (see Fig. 3). The topography is from the digital elevation model of SRTM15_PLUS^37^.

**Figure 2:**
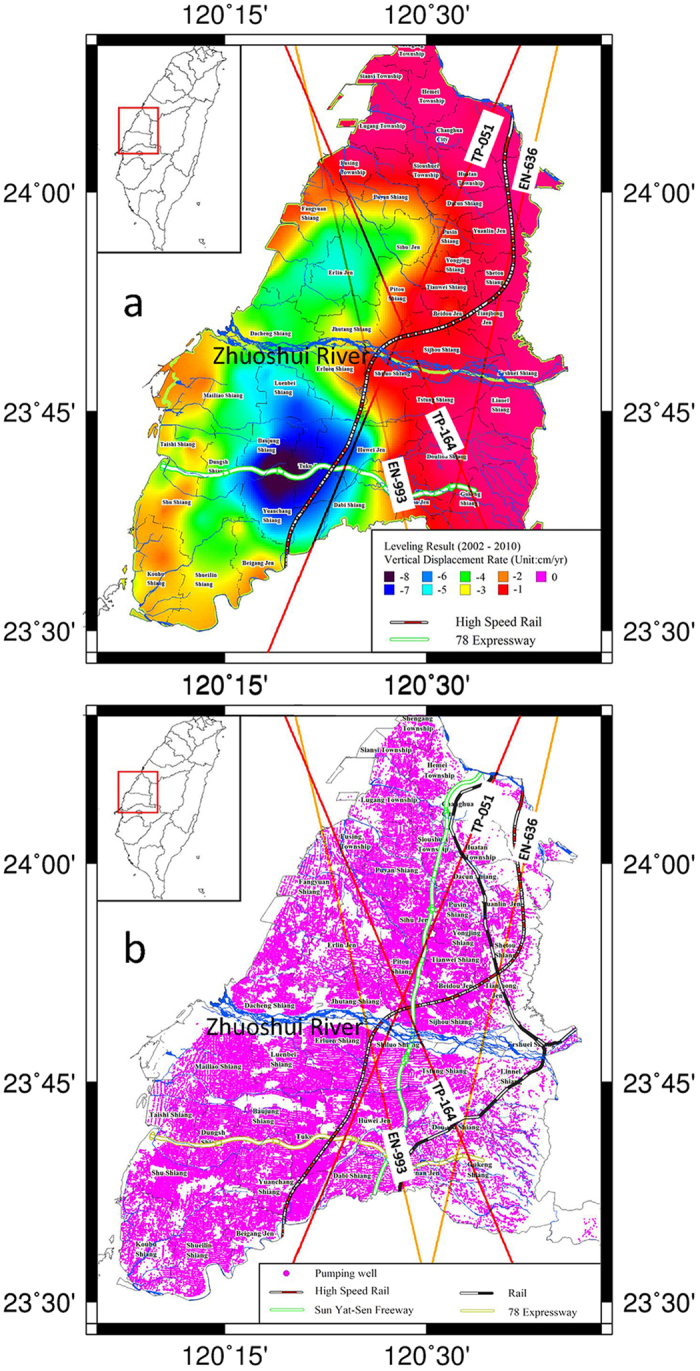
(**a**) Vertical displacement rates, from precision leveling in central Taiwan (Section S2, SI), (**b**) Distribution of about 310,000 groundwater pumping wells is in the same 2,364 km^2^ area as Fig. 2a. Overlapped are TOPEX/POSEIDON and ENVISAT satellite ground tracks (labeled as satellite abbreviation-pass number), where vertical displacement rates at a 1-km interval are derived from the two altimeters (see Fig. 3).

**Figure 3:**
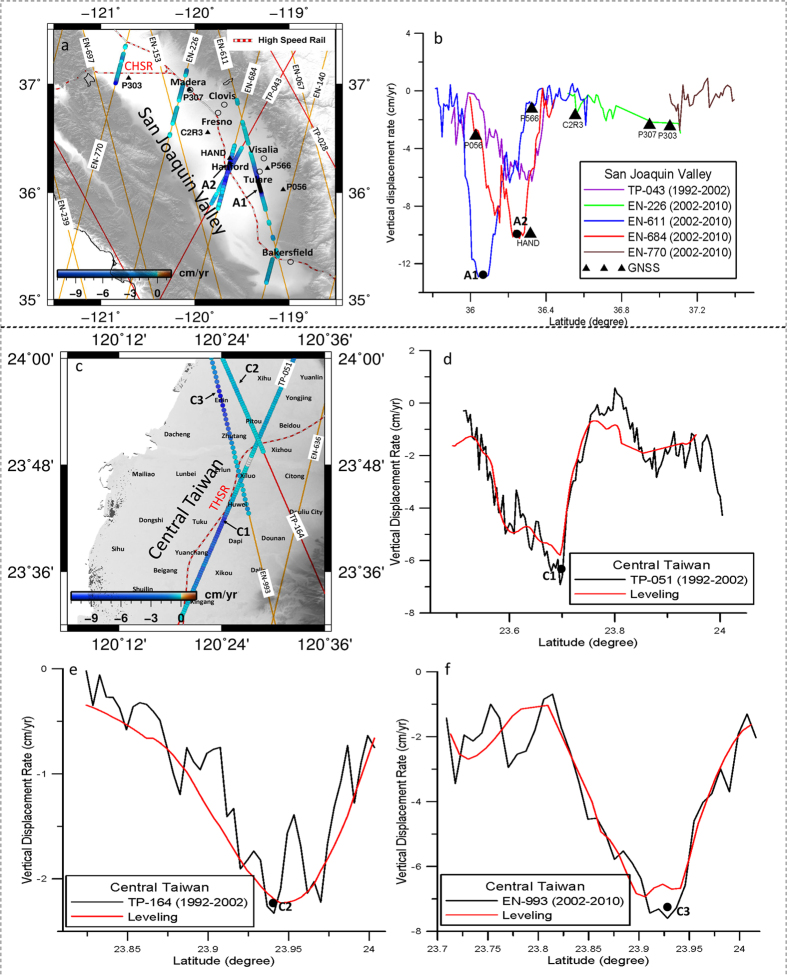
(**a**) Along-track subsidence rates from TP and EN in the San Joaquin Valley, (**b**) comparison between altimeter-derived rates and GNSS-derived rates in the San Joaquin Valley. A1 is the location of maximum subsidence from EN-611, A2 is the location of maximum subsidence from TP-043 and where accelerating subsidence is detected by TP, J1 and J2 over 1992–2015, (**c**) along-track subsidence rates in central Taiwan, (**d–f**) comparison with leveling-derived rates along TP-051, TP-164 and EN-993. C1 is the location of maximum subsidence in central Taiwan (see Fig. 2a), C2 and C3 are the centers of the bow-shaped subsidence patterns detected by TP-164 and EN-993. Note: negative vertical displacement corresponds to subsidence. GMT^36^ V5.1.3 is used to plot Fig. 3 (http://gmt.soest.hawaii.edu/). The topography is from the digital elevation model of SRTM15_PLUS^37^.

Subsidence rates derived from satellite radar altimetry (TP and EN) agree well with rates determined from precision leveling in CT and GNSS in the southern SJV (Fig. 3). Height changes derived from different radar altimetry missions also agree well. For example, in the SJV at the crossover of TP-043 and EN-684 (Fig. 3a), the time-series height changes derived from J1 (the follow-on mission for TP) and EN (Fig. S6, 2002–10) and the resulting subsidence rates (about 6 cm yr^−1^) show good agreement. Three major subsiding bowls identified in the SJV from the GNSS measurements (Fig. 1) are reflected in the TP and EN along-track subsidence profiles. Along TP-043, the largest altimeter-derived subsidence rate near Hanford (A2, Fig. 3a) in the central subsidence bowl is about 10 cm yr^−1^, and consistent with the GNSS-derived rates (Table S1). At GNSS stations P056 and P566, subsidence rates are <4 cm yr^−1^ and agree with the altimeter-derived rates to 1 cm yr^−1^. The largest subsidence rate from EN is ~13 cm yr^−1^ along EN-611 (A1, Fig. 3b). In CT, the two principal subsidence bowls identified by leveling (Fig. 2a) are reflected in the TP and EN along-track subsidence profiles, and the patterns and rates are consistent with those from leveling (Fig. 3d–f).

**Figure 4:**
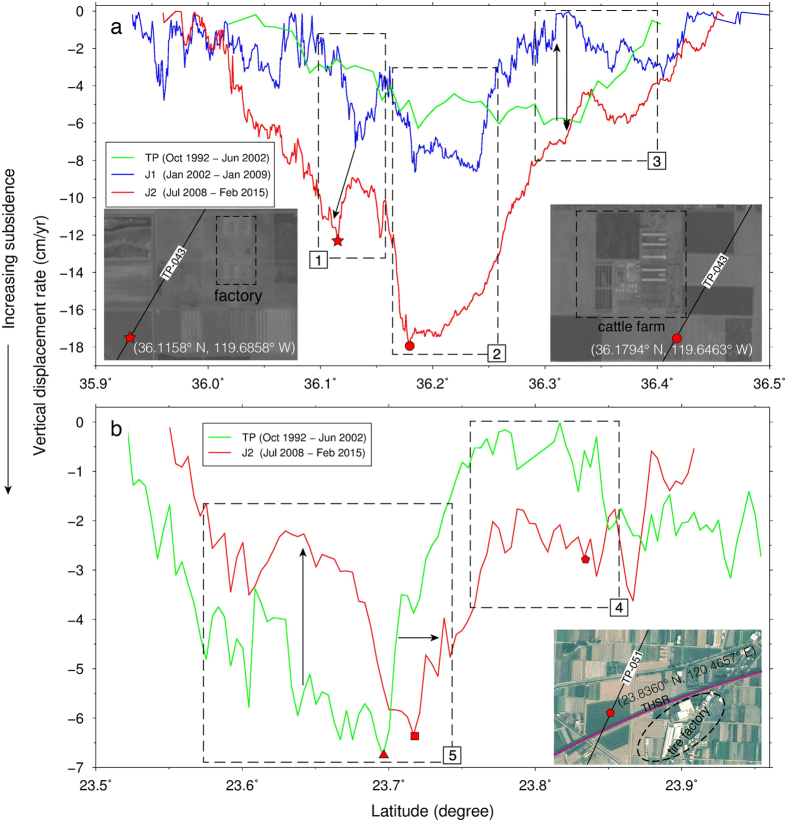
(**a**) Vertical displacement rates derived from TP, J1 and J2 over 1992–2015 in the San Joaquin Valley along TP-043. Inserted are two Landsat satellite images: the left image shows the region with an anomalous change of subsidence marked by the red star along TP-043 in Box 1 (dashed line). Box 2 shows the region with the maximum subsidence (the cumulative subsidence at red dot is 206 cm over 1992- 2015). The right image shows a cattle farm around the red dot. Box 3 shows an area with varying rates. (**b**) vertical displacement rates in central Taiwan along TP-051. The J1-derived rates contain large uncertainties in central Taiwan, and are not shown here. Inserted is a SPOT satellite image showing a tire factory in Box 4 (courtesy of Center for Space and Remote Sensing Research, National Central University). Box 5 covers the major subsidence feature. GMT^36^ V5.1.3 is used to plot Fig. 4 (http://gmt.soest.hawaii.edu/). The Landsat images were provided by: U.S. Geological Survey, Earth Resources Observation and Science (EROS) Center, 2015, Landsat products and services: EROS, Glovis Web page, accessed 29 September 2015 at http://glovis.usgs.gov/.

**Figure 5:**
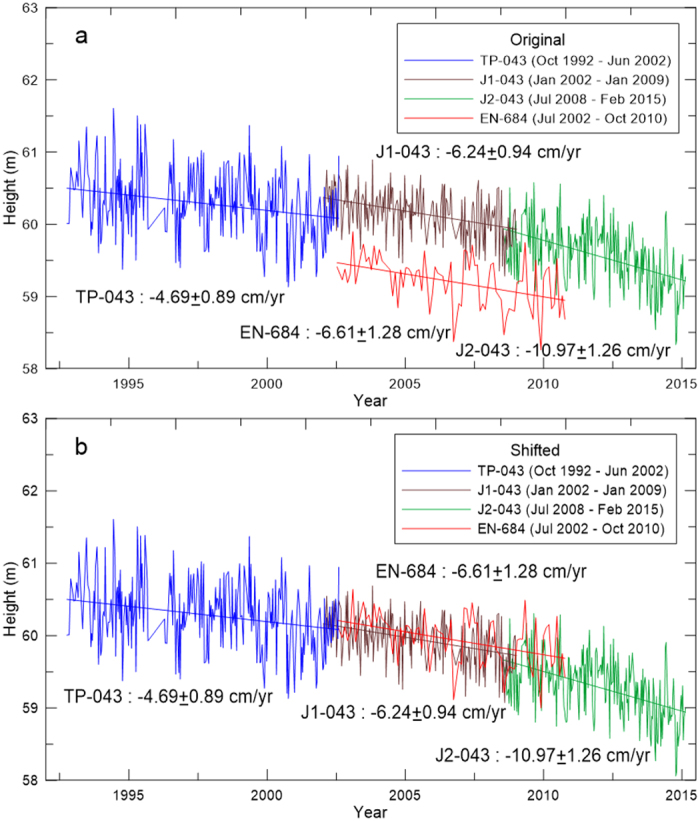
Height changes at the location of the maximum subsidence measured (in this study) in the San Joaquin Valley near Hanford, California along TP-043 (red dot in Box 2, Fig. 3a) from (**a**) the original heights and (**b**) the shifted heights.

Subsidence patterns in the SJV and CT are time-varying and could be influenced by changing groundwater use related to land-use, climatic and regulatory factors. Figure 4 shows patterns in the VDRs along profiles near Hanford in the SJV and in Tuku Township, CT (Fig. S7, Sec. S4, SI). In Box 1 (Fig. 4a), the VDRs derived from TP, J1 and J2 for the SJV show a consistent pattern from J1 and J2: a steep drop (increased subsidence rate) followed by a bump (decreased subsidence rate) in the profile. The drop in Box 1 reaches its lowest point (maximum subsidence rate) at 36.1158° N, 119.6858° W (red star, Fig. 4a). In Box 2 (Fig. 4a), the subsidence extent is expanding and the rates are accelerating during the period covered by the altimetry. At the location of maxium subsidence (Fig. 5), the VDR during Oct 1992–Jul 2002 is 6 cm yr^−1^, increasing to 8 cm yr^−1^ during Jan 2002–Jan 2009, and to 18 cm yr^−1^ during Jul 2008–Feb 2015. The increased groundwater pumping owing to the recent ongoing drought in California may partially contribute to the increased rate from J2. The subsidence rates in the SJV are time-varying: in Box 3 (Fig. 4a), initially the rates are about 6 cm yr^−1^ (TP), decrease to 3 cm yr^−1^ (J1), and increase to 7 cm yr^−1^ (J2).

The rates in CT also vary with time and space. In Box 4 (Fig. 4b), the subsidence rates increased in recent years. In Box 5 (Fig. 4b), between 23.60°N and 23.75°N, the changes in the subsidence rates from TP to J2 show the extent of subsidence shrinks (from green lines to red lines) in recent years. The location of maximum subsidence shifts northward to about 23.72°N and the extent of subsidence increases northward from 23.71 to 23.85°N. The shifts in patterns likely are due to measures restricting groundwater use along a section of THSR near the point of maximum subsidence (Figs S7 and S8).

Land subsidence can cause angular deflection (AD) of the THSR owing to vertical displacements of the individual structural support columns of the elevated railway. A large AD could weaken the foundational support of the railway and result in serious operational problems if not adequately mitigated^4^. Along the THSR section in Yunlin County (Fig. 2a), heavy groundwater pumping and heterogeneity of sediments with varying degrees of compressibility have led to large ADs, which once approached the 1/1000 limit set by the safety code. TP-051 is nearly parallel to THSR near Tuku (see Fig. S7) and altimetry data have already provided valuable observations to monitor ADs here. The planned California High-Speed Rail (CHSR) now faces the same potential risk of large ADs as the TSHR does: it passes through a region that has been experiencing subsidence due to heavy groundwater depletion. As it happens, EN-684 and TP-043 are perpendicular to the planned route of CHSR near Hanford and the observations from EN and TP follow-on missions can provide an effective means to monitor the subsidence as well as the ADs in the future.

**Figure 6:**
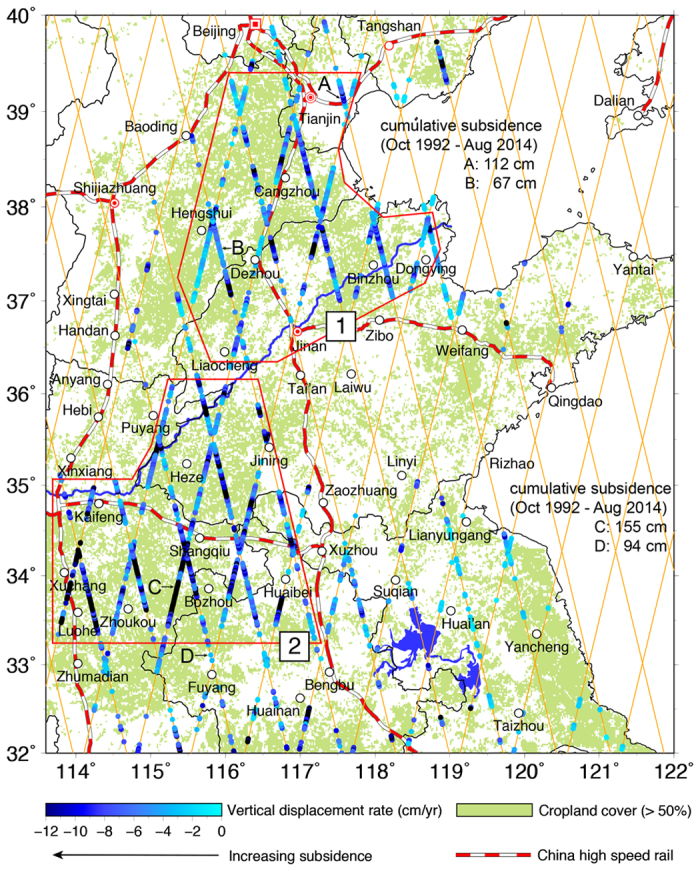
Vertical displacement rates derived from ENVISAT (Jul 2002–Oct 2010) where croplands constitute greater than 50% of land use in the NCP. The cropland cover is from Global Land Cover (GLC)-SHARE^38^, beta-release 1.0. Red symbols represent Beijing, Tianjin, Jinan and Shijiazhuang and empty circles represent other major cities. Rates with standard error greater than 5.00 cm yr^−1^ or with a signal-to-noise ratio <1.50 were rejected. Along-track locations without rates are over foothills, such as eastern Shandong Province. Two regions of major cropland subsidence (greater than 2 cm yr^−1^) are within the red-lined polygons. Letters A-D show the locations of TP-EN-J2 time series of height changes in Figs S11–14, with the cumulative subsidence values given. GMT^36^ V5.1.3 is used to plot Fig. 6 (http://gmt.soest.hawaii.edu/).

**Figure 7:**
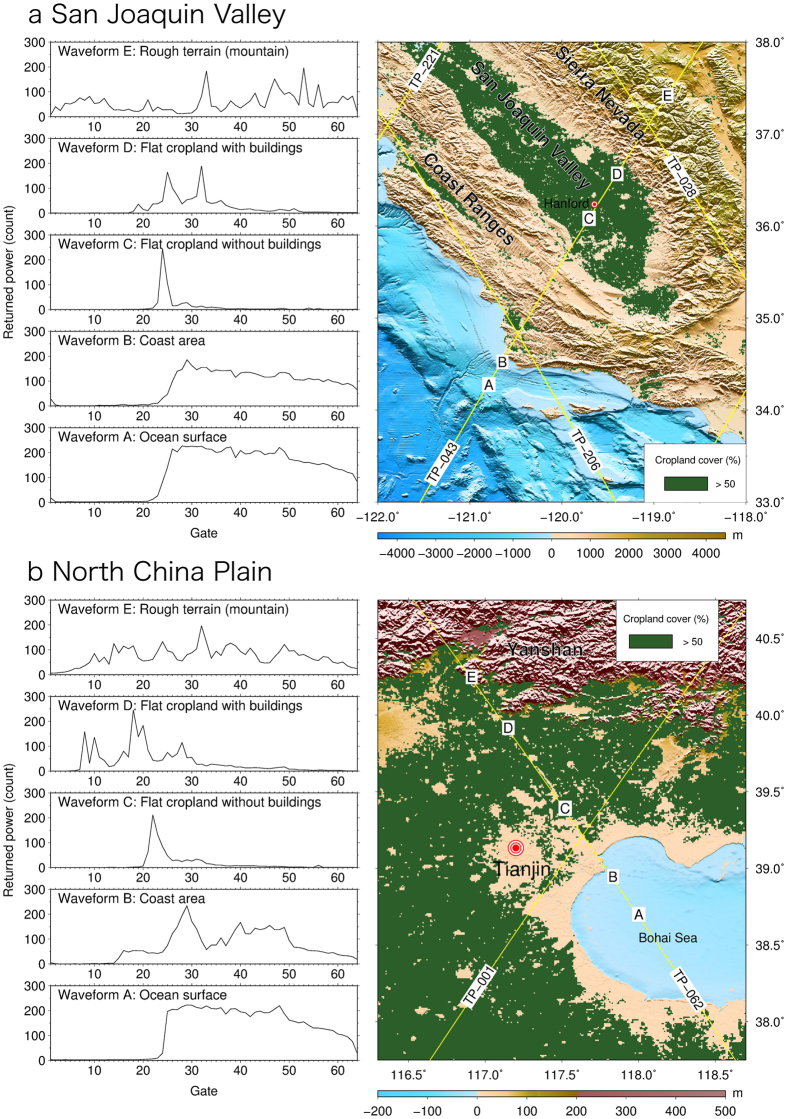
(**a**) TP altimeter Waveforms A–E over different surface types along Pass 043 (TP-043), covering the croplands of the San Joaquin Valley, (**b**) Waveforms A–E along TP-062 over the croplands of the North China Plain. The topography is from the digital elevation model of SRTM15_PLUS^37^, and the cropland cover is from Global Land Cover (GLC)-SHARE^38^, beta-release 1.0. Radar altimetry with Waveform C produces accurate vertical displacement rates (less than 1 cm yr^−1^) in flat lands with more than 50% crop cover. GMT^36^ V5.1.3 is used to plot Fig. 7 (http://gmt.soest.hawaii.edu/).

**Figure 8:**
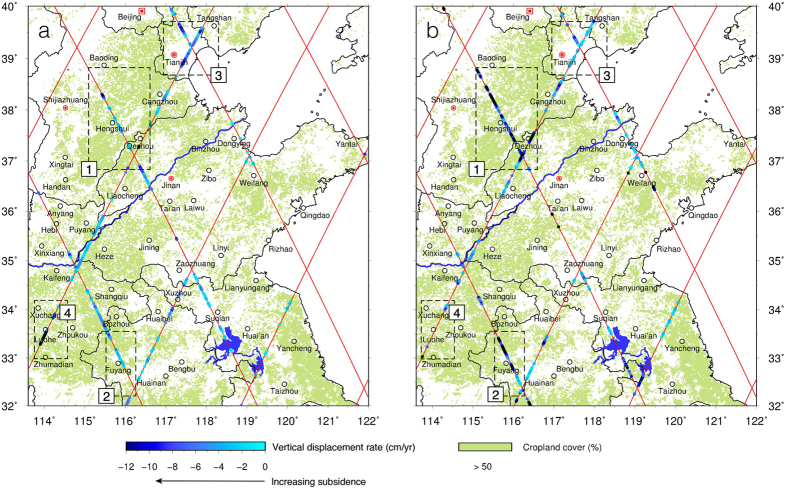
Vertical displacement rates in the croplands of the North China Plain from (**a**) TP (Aug 1992 – Jun 2002) and (**b**) J2 (Jun 2008 – Aug 2014). The cropland cover (shaded areas) is from Global Land Cover (GLC)-SHARE^38^, beta-release 1.0. GMT^36^ V5.1.3 (http://gmt.soest.hawaii.edu/) is used to plot the land cover, ground tracks and all the features.

EN identifies two regions of major cropland subsidence in the NCP (Fig. 6, also Figs S9–12): Region 1 covers eastern Hebei Province and northern Shandong Province, with an affected area of 67,900 km^2^; Region 2 covers eastern Henan Province and northern Anhui Province, with an affected area of 78,000 km^2^. In the foothills of the NCP, VDRs derived from EN and TP, and J2 are indeterminate because the altimetry waveforms are corrupted (see Waveform E, Fig. 7). The J1 rates contain large uncertainties in much of the NCP and are not shown here. TP and J2 detect time-varying subsidence elsewhere in the NCP (Fig. 8). For example, in Boxes 1 and 2 (Fig. 8), located in the Hebei and Anhui provinces, the along-track rates increased dramatically from the time of TP to the time of J2. In Box 3 (Fig. 8), near Tianjin, the J2 subsidence rates are less than those from TP, suggesting that groundwater extraction was reduced here due to measures implemented to control subsidence since the start of high-speed rail operations (Fig. S9). In Box 4 (Fig. 8), many VDRs were derived from TP, but only a few from J2. This is attributed to urbanization around this region, where new man-made structures (at the time of J2) contaminated altimeter waveforms and degraded ranging accuracies (see Waveform D, Fig. 7). The altimeter results identify many subsidence-affected areas and changing subsidence patterns in the NCP that are consistent with the temporal and spatial groundwater storage changes detected by GRACE^22^. The results can be used as guides for future, more detailed, precise geodetic and geotechnical measurements.

## Discussion

The causes for subsidence are complicated and not well understood in the study regions (SJV, CT and NCP). Groundwater abstraction may be a major contributing source for land subsidence. Near the point with the largest subsidence in SJV, operations in factory and cattle farms, such as those shown in Box 1 and 2 in Fig. 4a, may contribute partially to the steep subsidence observed along the profiles through abstraction of large amounts of groundwater. Along the THSR in CT, heavy groundwater extraction from surrounding factories, such as a tire factory (Fig. 4b), may be responsible for land subsidence. However, more studies are needed to investigate the roles of the farms and factories on the land subsidence detected in this paper.

Long-term, time-varying land subsidence over croplands from 1992 to present has been determined by TP, J1, J2 and EN altimeters using a dedicated processing method for land altimeter data. The result in the NCP suggests that a mission with a long repeat period and relatively small cross-track spacing (<50 km) like EN can measure cropland subsidence over a large area. The application of altimetry to subsidence monitoring can be extended globally to other croplands, especially in areas where terrestrial-geodetic subsidence monitoring methods may be lacking. The GNSS and leveling-derived vertical displacement rates in the SJV and CT are used to assess the accuracies of satellite altimetry, which are better than the one cm yr^−1^ level. Current altimetry technologies can detect subsidence at a 1-km along-track spatial resolution (c.f. Methods for details), and likely will improve as the technologies evolve. Combined use of leveling, GNSS, InSAR and radar altimetry methods for measuring, mapping and monitoring surface deformation and subsidence in particular, would optimally leverage the strengths of each of the different techniques to best address the scientific needs.

## Methods

A satellite-borne radar altimeter measures the two-way time delay of the radar pulse which is used to compute the distance between the satellite and the Earth’s surface, and after computing the precise orbit of the satellite, the altitude of the surface can be computed. Repeat-period altimetry can be used to measure changes in altitude (height) or height changes of the surface. The ground tracks are re-visited by the satellite at regular time intervals of weeks, and within about ±1 km of the reference track at the equator. Figure 7a shows the altimeter waveforms from the Pacific Ocean off the coast of California eastward to the SJV and the Sierra Nevada. The same evolution of waveforms is also demonstrated for the NCP (Fig. 7b). The Brown waveforms over the ocean (Waveform A, Fig. 7) and coast (Waveform B, Fig. 7) may result in precise range measurements for marine applications, but waveforms on land may be too contaminated to recover precise ranges for detecting height changes.

The waveform over flat terrain with crops (Waveform C, Fig. 7) or with snowpack, e.g., land regions near Hudson Bay, Canada^29^,^30^,^31^ can be retracked to yield a precise range change, after correcting surface gradients using the collinear track analysis. The surface roughness of croplands diffuses radar pulses, similar to wind-induced, small-scale waves in oceans. The surface of a fallow, flat and building-free cropland is similar to a calm lake surface—the altimeter waveform is specular with a steep leading edge compared to other types of waveform. In summer, the cropland surface roughens as crops grow, resulting in a waveform with a less steep leading edge. Similar to a significant wave height at sea, terrain undulation creates a ranging bias and modulates the footprint size of a pulse-limited radar. For example, a terrain undulation of 1 m at a length scale of several km leads to a pulse-limited footprint radius of about 1 km for EN and slightly larger for the TP series altimeters^28^,^29^ (pulse width = 3.125 ns). However, unlike significant wave height at sea, terrain undulation at a given location can remain constant for the satellite mission period, and the undulation-induced ranging bias will be eliminated when heights from repeat cycles are differenced.

Here we adopted a modified, enhanced version of the repeat-track method^32^,^33^,^34^ for the space and time reduction of raw height measurements from TP, J1, J2 and EN. For EN, we also estimated parameters that account for the effects of backscatter and the slopes of the leading and trailing edges^32^,^35^. For all missions, we chose to compute VDRs for bins spaced at about 1 km along satellite ground tracks. A bin is a circular region with a given radius, centered at a location along the mean ground track of all repeat cycles in a satellite mission. Over a flat cropland with a moderate undulation of about 1 m, the radius of an altimeter footprint is about 1 km. Within a given bin, all the raw height measurements (the sampling rate is 18 Hz for EN, and 10 Hz for TP, J1 and J2) from the repeat cycles were least-squares fitted to the following space and time function^3^:


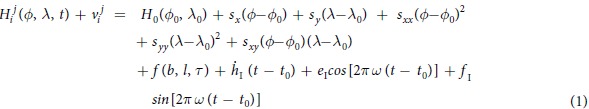


where


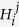
: height from altimeter, with range correction from the subwaveform threshold retracker (see below).

*j*: repeat cycle.

*i*: the *i* th measurement from repeat cycle *j* in the bin.

*ϕ*_0_, *λ*_0_: geodetic latitude and longitude of the bin center

*ϕ*, *λ*, *t*: geodetic latitude and longitude and time of 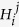


*t*_0_: reference time.

*H*_0_(*ϕ*_0_, *λ*_0_): mean height at the bin center for all repeat cycles.


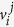
: residual of 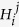.

*s*_*x*_, *s*_*y*_, *s*_*xx*_, *s*_*yy*_, *s*_*xy*_: coefficients of a 2^nd^ order surface fitting of the heights 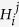 from all repeat cycles within a radius centered at the bin.


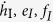
: initial rate of height change and initial annual terms used to improve the 2nd order surface fitting.

*b*, *l*, *τ*: effects of backscatter and the slopes of the leading edge and trailing edge of waveform on 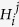.

*f* (*b*, *l*, *τ*): a function to model the effects *b*, *l*, *τ*. Note that these effects are not modeled in the waveforms of TP, J1 and J2 because they are not available.

The function *f* (*b*,*l*,*τ*) in Eq. (1) is expressed as


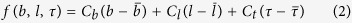


where 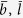 and 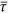 are the means of *b*, *l* and *τ* from all repeat cycles in the bin, and *C*_*b*_,*C*_*l*_ and *C*_*t*_ are the parameters that adjust these effects. For EN, up to 12 parameters could be estimated (see Table S2). We used a robust least-squares estimator, which is resilient against outliers, to estimate the parameters in Eq. (1) as follows. The first-round estimated parameters used all height measurements. Then, the residuals of the measurements and the standard deviations of the residuals were computed. We then used the three-σ outlier threshold to remove anomalous heights: if a residual 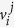 is three times larger than its standard deviation, the corresponding measurement 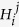 was removed, and the next-round parameter estimation was carried out. The final parameters were estimated from the measurements that pass the three-σ testing. Also, if the standard deviation of the residuals in a bin were larger than 5 m, the vertical rate in the bin was not computed.

Then, the space and time corrected 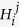 was computed as (the time correction accounts for the *b*, *l*, *τ* effects and is for EN data only)


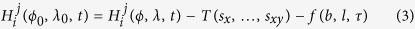


where *T* is the sum of terms 2–6 in Eq. (1) and 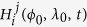 is regarded as a corrected height at the bin center at time *t*. For a given cycle *j*, we used the three-σ threshold to remove anomalous values 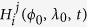 in the bin, and then compute the representative height for cycle *j*:


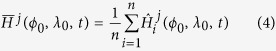


where 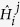 is an acceptable height measurement and *n* is the number of such measurements. With 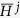 computed for all repeat cycles in the given bin (the mean position is at *ϕ*_0_, *λ*_0_), a time series of height was formed and least-squares fitted to the function


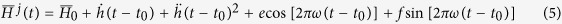


where 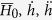 are the mean, VDR and vertical acceleration, and *e* and *f* account for the seasonal variation of height. Again, we used the robust least-squares estimator to determine the parameters in Eq. (5). Note that the terms 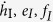 in Eq. (1) are just the initial estimates for VDR and annual variations; the final VDR is 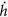 and the amplitudes of the annual variations are *e* and *f*. In Sec. S3 of Supplementary Information (SI), we selected an optimal waveform retracker to recover precise ranges from TP, J1, J2 and EN from waveforms like Waveform C in Fig. 7 for the SJV and NCP. Note that backscatter effects are not considered for TP, J1 and J2 in this study because of a lack of the necessary data.

The repeat-track method also diminishes the anisotropy roughness effect due to non-circular radar polarization, which causes different radar echoes for descending and ascending passes for the EN altimeter system. In fact, such height measurements do not repeat the exact same locations (maximum lateral offset: 1 km) and the measurements are affected by a number of time-varying factors. As a summary, the following conditions should be met to obtain precise heights on land: (a) sufficiently flat terrain within the altimeter footprint that may be covered with short vegetation, such as crops, (b) low percentage of buildings in the footprint area, (c) use of a proper waveform retracker, and (d) proper accounting of radar backscatter effects using an empirical threshold value. Parts of the croplands in the SJV, CT and the NCP meet conditions (a,b). If the proportion of cropland within a “bin” of selected and processed data is greater than 50%, the accuracy of the resulting altimeter-derived VDR is generally better than 1 cm yr^−1^ (at 1-km resolution), based on the assessments using leveling data (CT, Table S3) and crossover differences (CT and NCP, Table S4).

Typically, there will be inter-mission altimeter range biases between two altimeter missions, and such biases depend on surface attributes (ocean, land, ice and river, etc). The combination of inter-mission altimeter bias and terrain-induced height difference between reference tracks will lead to an apparent discontinuity between the two time series of height changes from two satellite missions at the crossovers of two ground tracks (Fig. S6). Because the VDRs in this study are computed for individual satellites and are the time derivatives of heights, the inter-mission discontinuities will not affect individual rates. In this study, we compute the cumulative subsidence, *S*, at a given bin from multiple satellite missions, such as TP, J1 and J2, using


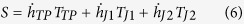


where 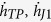 and 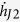 are the mean rates from TP, J1 and J2, and *T*_*TP*_,*T*_*J*1_ and *T*_*J*2_ are the time spans over which rates are computed. For example, at the locations corresponding to Figs 5 and S6, the cumulative subsidence values from Oct 1992 to Feb 2015 are 206 cm and 153 cm, respectively.

Given a height time series (see Figs 5 and S6, S9–S12), both the VDR and its uncertainty (one-standard deviation, at the 68% confidence level) are computed (see Eq. S5), and they can be used to determine the rate’s signal-to-noise ratio for decision making. Because of the influence of height oscillations (the key component is annual oscillation), the uncertainty of a VDR tends to decrease with the length of time series. As such, the altimetry method in this paper would be more applicable to long-term (longer than one year) subsidence monitoring.

## Additional Information

**How to cite this article**: Hwang, C. *et al*. Time-varying land subsidence detected by radar altimetry: California, Taiwan and north China. *Sci. Rep.* **6**, 28160; doi: 10.1038/srep28160 (2016).

## Supplementary Material

Supplementary Information
