# Supplementary material for: Time-varying land subsidence detected by radar altimetry: California, Taiwan and north China
Source: Sci Rep. 2016 Jun 21;6:28160. doi: 10.1038/srep28160 (PMC4914853; doi:10.1038/srep28160)
Supplement: Supplementary Information [file srep28160-s1.doc]

**Supplementary information**

**Time-varying land subsidence revealed by radar altimetry: California, Taiwan and north China**

Cheinway Hwang^1,*^, Yuande Yang^2^, Ricky Kao^1^, Jiancheng Han^1^, C.K. Shum^3,7^, Devin L. Galloway^4^, Michelle Sneed^5^, Wei-Chia Hung^6^, Yung-Sheng Cheng^1^, and Fei Li^2^

^1^Department of Civil Engineering, National Chiao Tung University, 1001 Ta Hsueh Road, Hsinchu, Taiwan.

^2^Chinese Antarctic Center of Surveying and Mapping, Wuhan University, 129 Luoyu Road, Wuhan 430079, China.

^3^State Key Laboratory of Geodesy and Geodynamics, Institute of Geodesy & Geophysics, Chinese Academy of Sciences, Wuhan 43077, China

^4^U.S. Geological Survey, 5957 Lakeside Blvd, Indianapolis, IN 46278, U.S.A.

^5^U.S. Geological Survey, 6000 J Street, Placer Hall, Sacramento, CA 95819, U.S.A.

^6^Green Environmental Engineering Consultant Co. LTD, Hsinchu, Taiwan.

^7^Division of Geodetic Science, School of Earth Sciences, the Ohio State University, Columbus, OH 43210, U.S.A.

^*^Corresponding author: Cheinway Hwang, [cheinway@mail.nctu.edu.tw](mailto:cheinway@mail.nctu.edu.tw)

**S1: GNSS data showing land subsidence in the Central Valley, California and for validating vertical displacement rates derived from radar altimetry**

The vertical displacement rates (VDRs) in Fig. 1 over the Central Valley (includes the San Joaquin Valley [SJV] and the southern portion of the Sacramento Valley) and the surrounding areas are based on the measurements collected at the GNSS stations in the networks of CentralCalifornia, CentralCalifornia_SGPS, SFBayArea, SFBayArea_SGPS, NCalifornia, NCalifornia_SGPS, LongValley, and BasinAndRange_SGPS (http://earthquake.usgs.gov/monitoring/gps/). In total, there are 803 continuous and 696 campaign-mode stations used in this study. Table S1 shows the GNSS stations with the 15 largest measured subsidence rates over croplands in the SJV and the southern portion of the Sacramento Valley within the Central Valley. The first campaign-mode station was H104, established and first collected data in 1993. Note that a subsidence rate is the negative of a VDR and is a positive number indicating the downward movement of ground surface. Near the Hanford Municipal Airport (Fig. S1), the GNSS-derived subsidence rate is 9.79 ± 0.05 cm yr^-1^, which is the largest among the 1499 stations. Hanford is surrounded by flat croplands where satellite altimetry produces the most accurate and best coverage of VDRs measured in this study.

**Table S1:** Geodetic coordinates of the GNSS stations with 15 largest measured vertical displacement rates in the Central Valley (includes the San Joaquin Valley and the southern portion of the Sacramento Valley), and their horizontal velocities (north and east components). ‘*’ for Campaign-mode station; otherwise continuous station

| Station | Longitude (°) | Latitude (°) | North (cm yr^-1^) | East (cm yr^-1^) | Vertical (cm yr^-1^) | Time span |
| --- | --- | --- | --- | --- | --- | --- |
| P271 | -121.715 | 38.657 | -0.47 ± 0.03 | -2.32 ± 0.03 | -0.80 ± 0.03 | 6/2004-5/2015 |
| UCD1 | -121.751 | 38.536 | -0.31 ± 0.02 | -2.22 ± 0.02 | -1.14 ± 0.02 | 1/1999-5/2015 |
| RIOV^*^ | -121.694 | 38.149 | -0.50 ± 0.07 | -1.98 ± 0.08 | -1.12 ± 0.29 | 2/2002-1/2012 |
| P270 | -122.055 | 39.244 | -0.49 ± 0.03 | -2.24 ± 0.03 | -0.15 ± 0.03 | 5/2005-5/2015 |
| P273 | -121.388 | 38.116 | -0.29 ± 0.03 | -2.26 ± 0.03 | -0.32 ± 0.03 | 10/2005-5/2014 |
| P275 | -121.215 | 38.322 | -0.36 ± 0.03 | -2.28 ± 0.03 | -0.20 ± 0.03 | 7/2006-5/2015 |
| P303 | -120.705 | 37.054 | -0.19 ± 0.03 | -2.22 ± 0.03 | -2.43 ± 0.03 | 7/2005-5/2015 |
| P307 | -120.058 | 36.947 | -0.05 ± 0.03 | -2.48 ± 0.03 | -2.30 ± 0.03 | 10/2005-5/2015 |
| C2R3^*^ | -119.863 | 36.554 | -0.15 ± 0.04 | -2.23 ± 0.04 | -1.59 ± 0.10 | 6/2001-1/2014 |
| P566 | -119.229 | 36.324 | -0.17 ± 0.03 | -2.32 ± 0.03 | -1.12 ± 0.03 | 11/2005-5/2015 |
| P056 | -119.063 | 36.027 | -0.01 ± 0.03 | -2.36 ± 0.03 | -3.13 ± 0.03 | 11/2005-5/2015 |
| P565 | -119.237 | 35.744 | -0.04 ± 0.03 | -2.39 ± 0.03 | -1.77 ± 0.03 | 11/2005-5/2015 |
| ARM2 | -118.911 | 35.201 | 0.00 ± 0.03 | -2.65 ± 0.03 | -1.85 ± 0.03 | 12/2000-5/2015 |
| BKR1 | -119.109 | 35.132 | 0.26 ± 0.03 | -2.55 ± 0.03 | -1.77 ± 0.03 | 2/2004-5/2015 |
| HAND^*^ | -119.628 | 36.317 | -0.37 ± 0.03 | -2.50 ± 0.03 | -9.79 ± 0.05 | 10/2001-1/2014 |

The GNSS station coordinates and velocities refer to the North America Reference Frame, which is tied to the International Terrestrial Reference Frame 2008 (ITRF2008). The distribution of these stations is shown Fig. S1. Stations ARM2, P565, HAND, P307, P303, P273 and P275 are near the planned routes of the California High Speed Rail (CHSR).


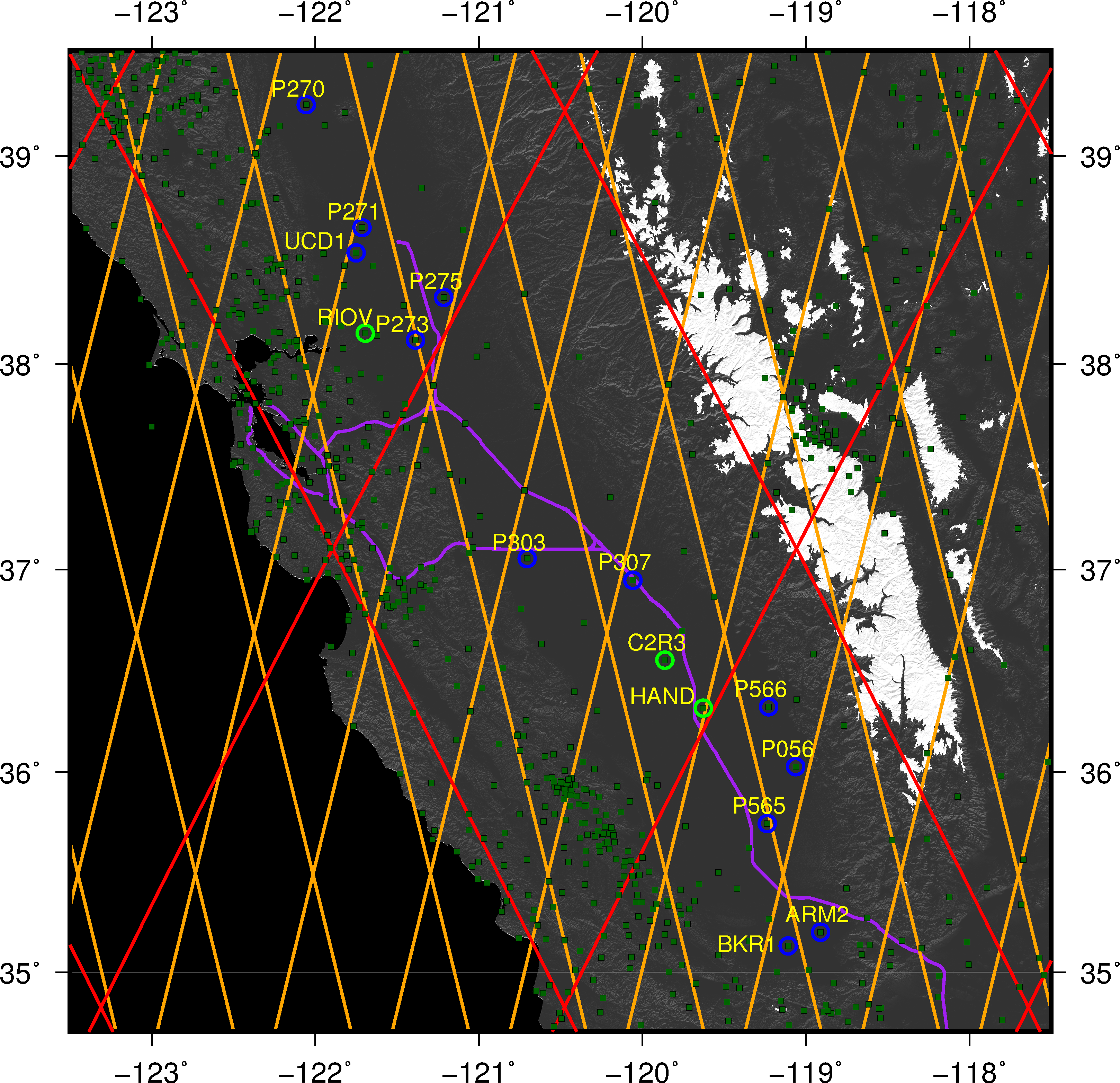


**Fig. S1:** The 15 GNSS stations with top subsidence rates in the Central Valley (includes the San Joaquin Valley and the southern portion of the Sacramento Valley) in Table S1, overlapped with the ground tracks of ENVISAT (orange) and TOPEX/POSEIDON (red), and the planned routes of the CHSR (purple), with different colors belonging to different construction stages. Dark green is all the GNSS stations in the Central Valley and around mountains. Blue and green points correspond to continuous GNSS stations and campaign-mode GNSS stations, respectively. (Background: low-resolution DEM from SRTM15_PLUS (ref. 31, main paper) plotted by the authors). GMT V5.1.3 (<http://gmt.soest.hawaii.edu/>) is used plot the DEM and tracks.

**S2: Leveling data showing land subsidence in central Taiwan and for validating vertical displacement rates from radar altimetry**

Fig. S2 shows the networks of leveling benchmarks, GNSS stations and compaction monitoring wells (borehole extensometers) in central Taiwan for subsidence monitoring. The funds to deploy all these sensors, collect differential heights from leveling and analyze the results are provided by the Water Resource Agency of Taiwan. The subsidence rates from this leveling network are presented in Fig. 2a and are used to validate the VDRs derived from altimeters (Section S3).

Since the early 1990s, the benchmarks in Fig. S2 have been surveyed annually using precision leveling with an allowable misclosure (double run) of 3$\sqrt{K}$ mm, where *K* is the distance between two neighboring benchmarks. We used careful field-work schedules to collect the leveling data to avoid (1) the aliased errors due to vertical motions of benchmarks during the times of observations (2) seasonal effect of ground water. A reference benchmark, called WR12, was established east of the leveling network in a region of no historical subsidence. We established a continuous GNSS station near WR12. The records at this GNSS station confirm that WR12 is indeed stable. The height differences between WR12 and the benchmarks in the network (Fig. S2) were measured to compute subsidence rates.

The subsidence rates derived from leveling are consistent with the VDRs from GNSS and compaction monitoring wells to about 1 cm yr^-1^ (Ref. 1). In 2014, 424 benchmarks were in the leveling network, and the total length of the leveling routes was 870 km. The number of benchmarks varies from year to year, but the variations are small. In some cases, we deployed densified, local leveling networks in areas of suspected large subsidence rates, e.g., where the Taiwan High Speed Rail (THSR) intersects Expressway No. 78. Here subsidence affects the safety of the THSR.


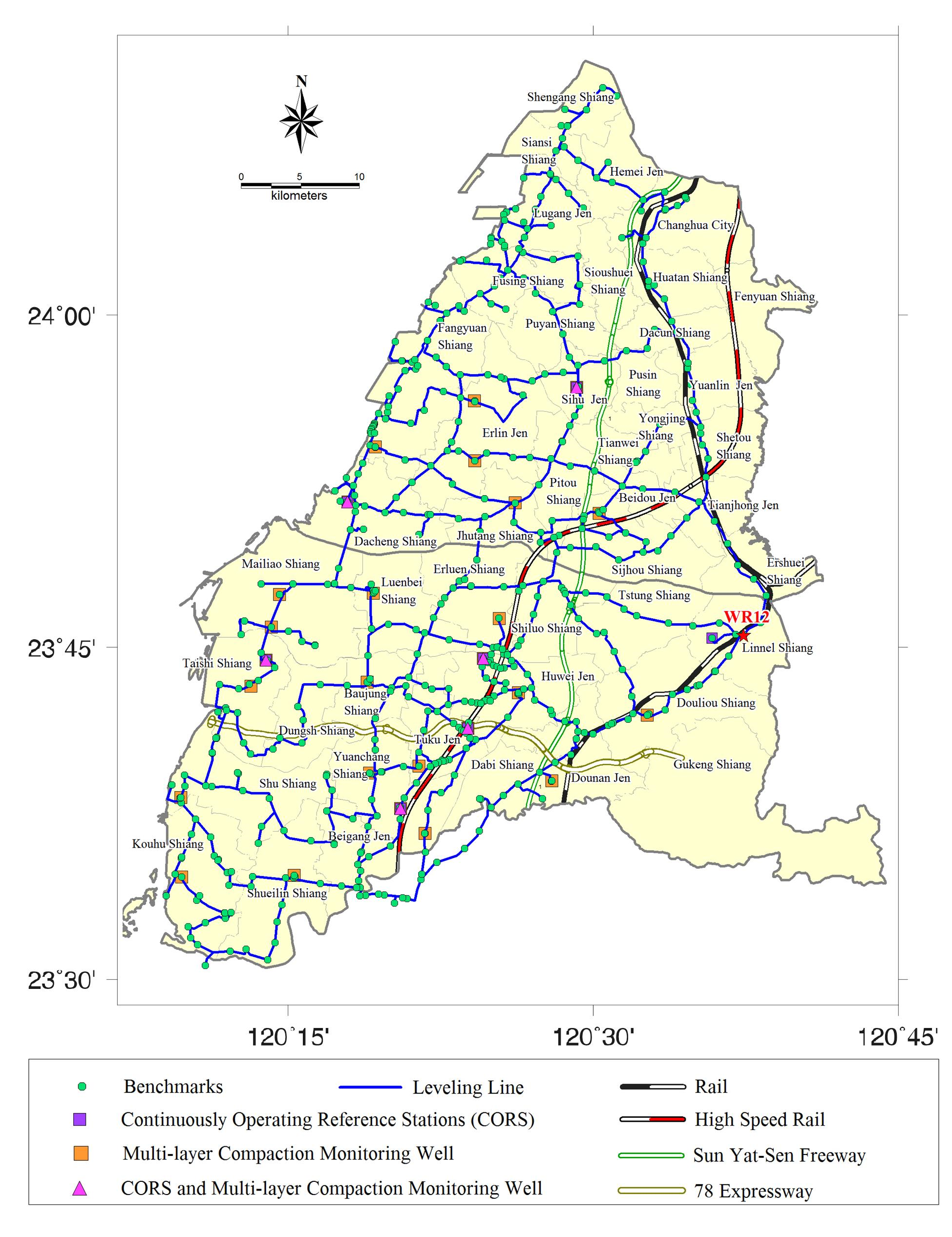


**Fig. S2:** Networks of leveling benchmarks, GNSS stations, compaction monitoring wells for monitoring land subsidence in central Taiwan. WR12 (star) is the reference benchmark. Vertical displacement rates from the leveling network are used to show the subsidence rates in Fig. 2a and to assess the altimeter-derived VDRs in Section S3. This figure is generated using MapInfo 10.0, <http://www.pitneybowes.com/us/location-intelligence/geographic-information-systems/mapinfo-pro.html>), using boundaries, roads and township names are from our databases.

**S3: Altimeter data and waveform retracking and accuracy assessment for altimeter measurements of land subsidence**

In the Methods section of the main paper, we have presented the key method of altimeter data processing for determination of vertical displacement rate. Here we provide more information. This section shows the altimeter data, and the method of retracking for obtaining acceptable rates of height change (VDRs) on cropland. The altimeter-derived VDRs are assessed by comparing to the Taiwan leveling results in Section S2. The geophysical data records (GDRs) and Sensor Data Records (radar waveforms) of TOPEX/POSEIDON (TP), JASON-1 (J1), JASON-2 (J2) are from AVISO (http://www.aviso.altimetry.fr) and those of ENVISAT (EN) are from European Space Agency. In the EN GDRs, range corrections based on the following retrackers are supplied: Ice1, Ice2, Sea ice and Ocean retrackers. However, as demonstrated below, we used range corrections from the subwaveform retracker^2^ to derive VDRs. The EN GDRs also supply backscatter power change and slopes of leading and trailing edges. No such range corrections and backscatter and leading/trailing edge effects are supplied in the GDRs of TP, J1 and J2. Like in oceans, geophysical corrections were applied to raw altimeter height measurements on land, including solid earth and pole tides, and to remove both the dry and wet tropospheric delays and the ionospheric effect of the altimeter range measurements.

For EN, it turns out that a proper selection of the three parameters in Eq. (2) is critical to obtaining good range accuracy (note: no need to have this selection for TP, J1 and J2 because of lack of the needed data in the GDRS of these satellites). To assess the impact of the three parameters, we experimented with 8 cases of parameter combination. Each case is associated with a binary (on or off) treatment of the three parameters. We then compared the resulting VDRs computed for the period 2002–2010 along EN Pass 993 (EN-993) with the VDRs from precision leveling (Section S2). EN-993 travels through a region of major subsidence in central Taiwan (Fig. 2a). The result of the rate comparisons for the 8 cases is given in Table S2. Fig. S3 shows the VDRs from leveling and from EN in the 8 cases. The differences can be further reduced by applying a filter to the original VDRs from EN. Fig. S4 compares the VDRs from leveling and from the filtered and unfiltered results of EN. The assessments using Table S2, and Figs. S3 and S4 suggest that the Case 4, 7 and 8 combinations of parameters in Eq. (2) result in similar accuracies in EN-derived VDRs of about 0.85 cm yr^-1^. Taking into account the current result of parameter combinations from peer groups^3, 4, 6^, we decided to use the Case 8 parameter combination for EN in the following experiments of waveform retracking. Again, we do not have data for modeling these effects for TP, J1 and J2.

**Table S2:** Statistics of the differences (in cm yr^-1^) between vertical displacement rates from EN and from leveling along EN-993 in central Taiwan using 8 cases of parameter combinations (0: parameter off; 1: parameter on).

| Case | Backscatter | Leading edge | Trailing | Differences | | | |
| --- | --- | --- | --- | --- | --- | --- | --- |
|  |  |  |  | Max | Min | Mean | RMS |
| 1 | 0 | 0 | 0 | 1.82 | -3.44 | -0.28 | 1.25 |
| 2 | 1 | 0 | 0 | 1.79 | -1.99 | 0.14 | 0.88 |
| 3 | 0 | 1 | 0 | 1.69 | -3.29 | -0.18 | 1.15 |
| 4 | 0 | 0 | 1 | 1.98 | 1.33 | 0.01 | 0.85 |
| 5 | 1 | 1 | 0 | 2.15 | -1.94 | 0.11 | 0.93 |
| 6 | 1 | 0 | 1 | 2.31 | -1.23 | 0.28 | 0.95 |
| 7 | 0 | 1 | 1 | 2.06 | -1.71 | 0.06 | 0.85 |
| 8 | 1 | 1 | 1 | 2.32 | -1.14 | 0.23 | 0.85 |


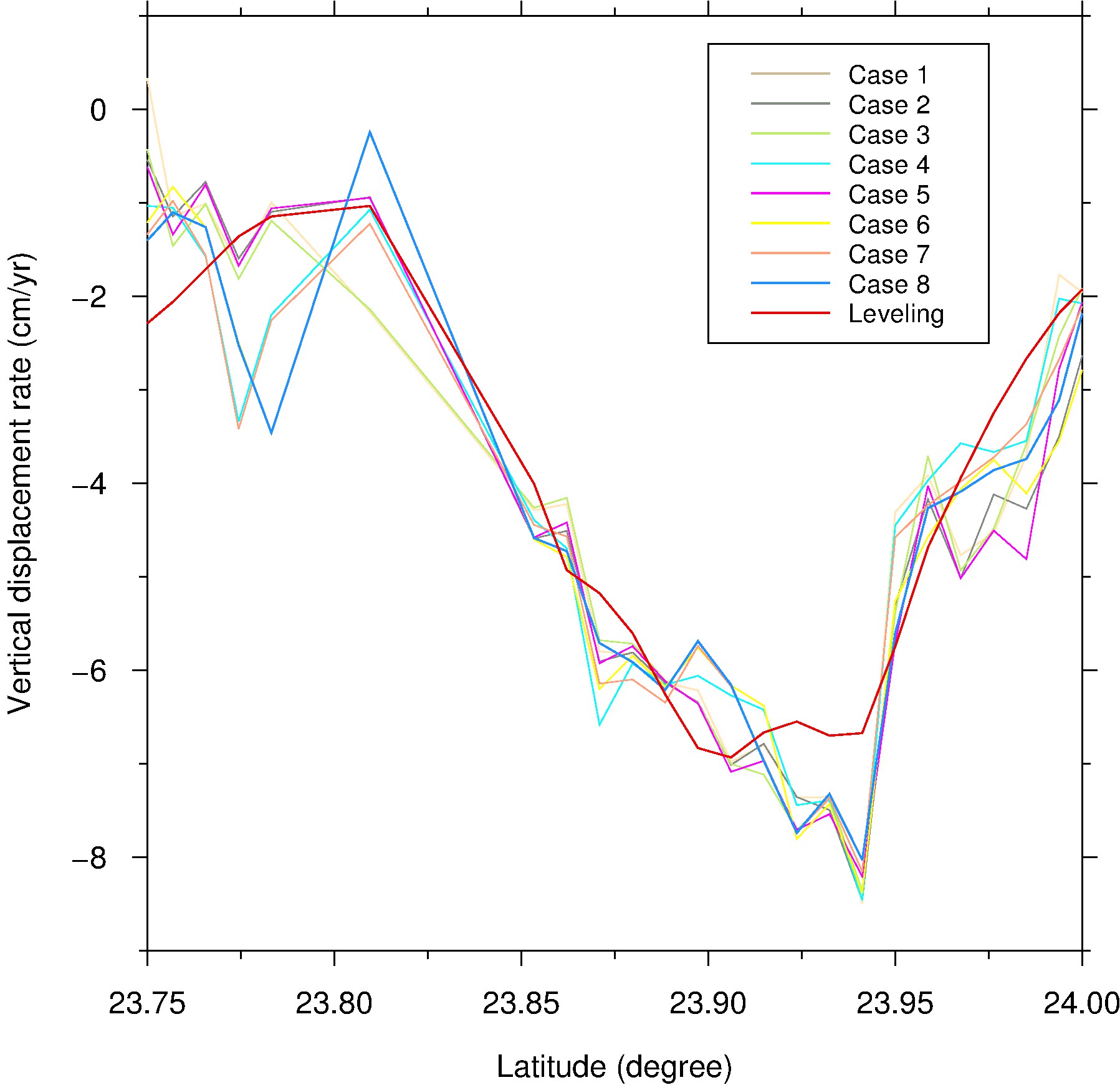


**Fig. S3:** Vertical displacement rates from precision leveling and from corrected heights of EN altimeter along Pass 993 (EN-993). For the 8 cases see Table S2.


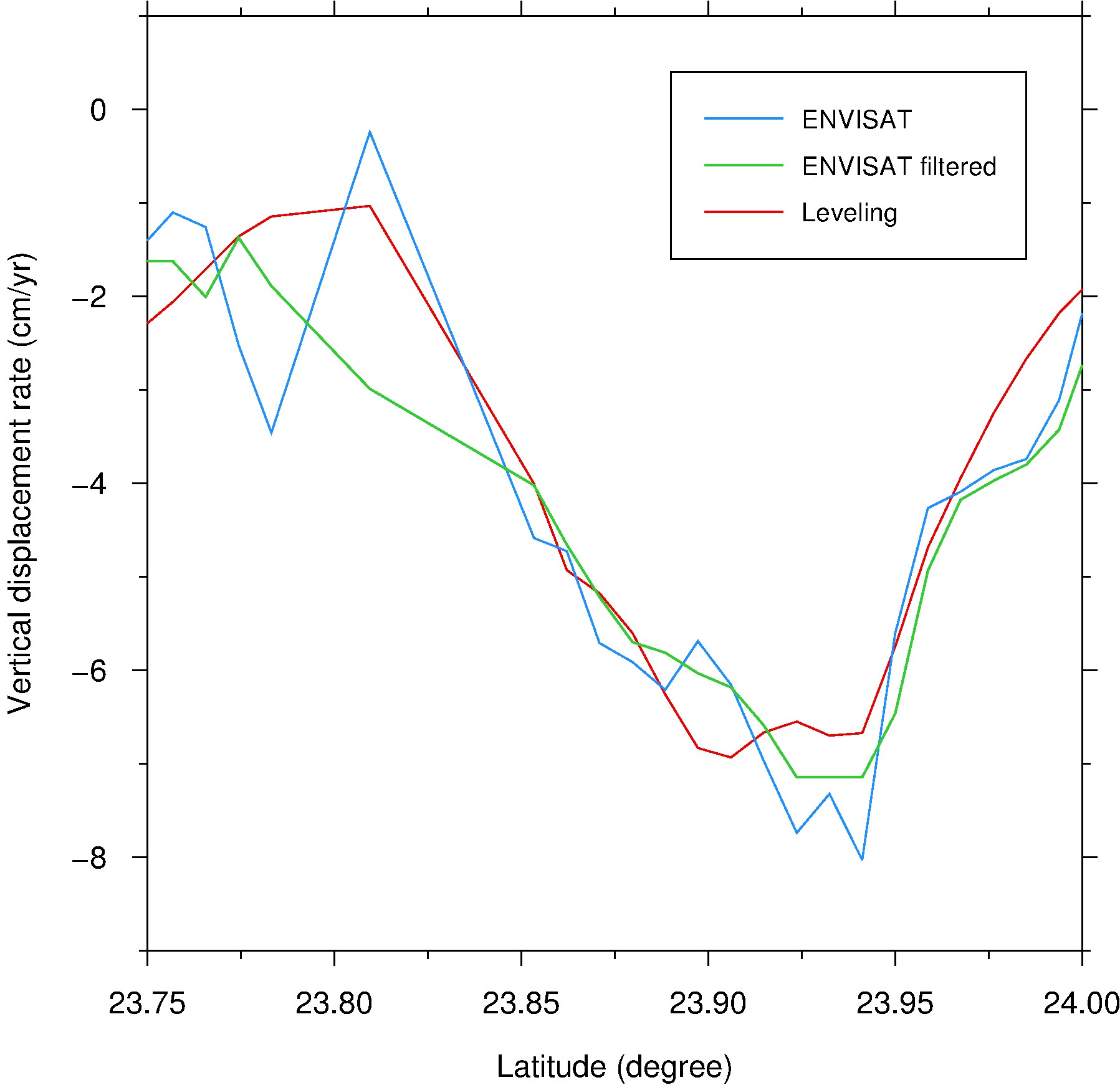


**Fig. S4**: Vertical displacement rates from leveling and from filtered and unfiltered results of EN altimeter along Pass 993 in central Taiwan.

In Eq. (1), heights $H_{i}^{j}$ are based on range corrections using the subwaveform threshold retracker^5^. Here we show that the subwaveform threshold retracker indeed outperforms other retrackers, based on comparisons between EN-derived VDRs and those from precision leveling in Taiwan (see Section S2). As shown earlier, the EN product supplies range corrections using the Ice1, Ice2, Sea ice and Ocean retrackers. In this study, the range corrections were also determined using the subwaveform threshold retracker and the offset center of gravity (OCOG) retracker^7^. The computational steps using the subwaveform threshold retracker are as follows^2^:

Step 1: Obtain a reference subwaveform from the full Brown waveform model.

Step 2: Compute the correlations between candidate subwaveforms and the reference waveform to find the optimal subwaveform.

Step 3: Determine the gate number,$G_{r}$, at the center of the leading edge from the optimal subwaveform by the improved threshold retracking with the optimal threshold value.

Step 4: Compute the retracking correction using $C_{R}=(G_{r}-G_{t})\Delta R$, where $\Delta R$ is the width of the radar pulse and $G_{t}$ is the theoretical gate number.

In Step 3, the optimal threshold value is a value that will remove the effects of radar penetration in soil and of sub-surface volume scattering of radar^8^. In this study, the optimal threshold value was determined by an empirical method as follows: we experimented with various threshold values to obtain range corrections and then compute VDRs. The computed VDRs were compared with the measured VDRs from leveling (Section S2), and the optimal threshold value was the threshold value that resulted in the least difference between the VDRs from leveling and altimetry. Table S3 shows the statistics of the differences between the VDRs from leveling and from EN in central Taiwan. The VDRs from EN are based on range corrections supplied in the GDRs and those from using various threshold values in the subwaveform threshold retracker. Fig. S5 shows the various VDRs. The results from Table S3 and Fig. S5 suggest that 0.10 is the optimal threshold value and results in a VDR accuracy of less than 1 cm yr^-1^. The altimeter-derived VDRs shown in Figs. 3, 4, 5, 6, 8 are based on corrections by the subwaveform threshold retracker using the 0.10 optimal threshold value.


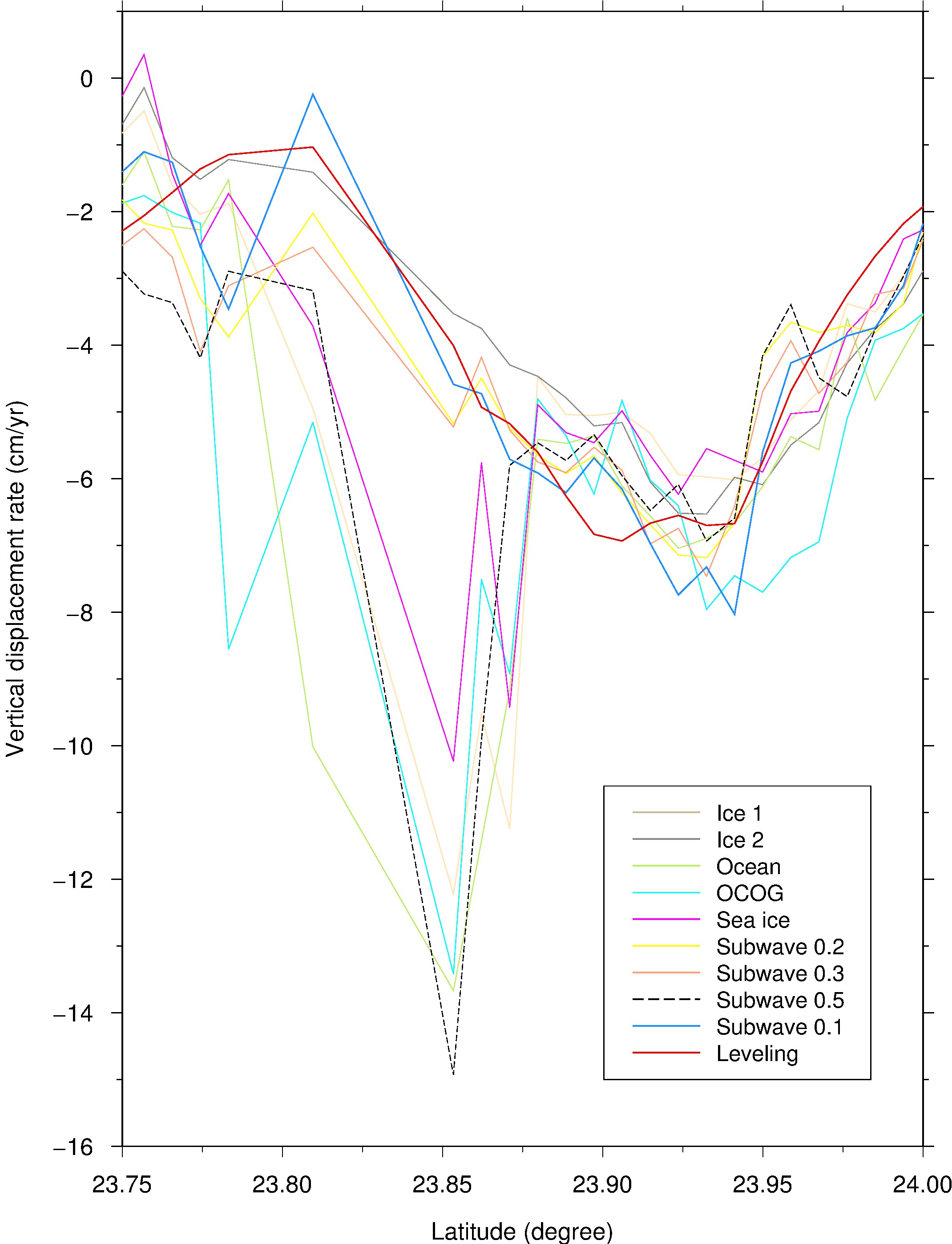


**Fig. S5:** Vertical displacement rates from leveling and from retracked height measurements along EN-993.

**Table S3:** Statistics of the differences between vertical displacement rates derived from leveling and from EN-993 by different retrackers in central Taiwan (unit: cm yr^-1^)

| Retracker | Max | Min | Mean | RMS |
| --- | --- | --- | --- | --- |
| Subwave^1^ 0.1^2^ | 2.32 | -1.14 | 0.23 | 0.85 |
| Subwave 0.2 | 2.73 | -1.58 | 0.14 | 0.97 |
| Subwave 0.3 | 2.73 | -1.30 | 0.37 | 0.99 |
| Subwave 0.5 | 10.91 | -1.69 | 0.86 | 2.54 |
| Ice 1^3^ | 8.22 | -1.93 | 0.65 | 2.40 |
| Ice 2^3^ | 2.01 | -1.92 | -0.11 | 1.04 |
| Sea ice^3^ | 6.22 | -2.41 | 0.34 | 1.82 |
| Ocean^3^ | 9.66 | -1.48 | 1.46 | 3.05 |
| OCOG | 9.41 | -2.11 | 1.46 | 2.91 |

^1^subwaveform threshold retracker

^2^threshold value

^3^ retrackers supplied in EN GDRs

If there is no leveling data over a study region, one can use differences between the VDRs at the crossover of two intersecting ground tracks (one ascending and one descending track) to assess the altimeter-derived rates. However, one must be cautious in choosing the bin radius to estimate the parameters for the space and time reductions needed in Eq. (1). Also, the slopes of the terrain near the crossover along the two tracks can vary, resulting in different slope-induced effects that are difficult to correct. Unless the area near a crossover is sufficiently flat and the measurements are dense, it is difficult for the ascending and descending tracks to produce equally good parameters for data reductions and equally good VDRs from the two tracks.

Despite the difficulty in using the crossover approach for assessing altimeter results, here we show two examples of crossover assessment using the VDRs derived from TP-051 (an odd pass number indicates an ascending track) and TP-164 (an even pass number indicates a descending track) in central Taiwan, and the rates derived from TP-001 and TP-062 near Tianjin, northern China. Table S4 shows the differences between the rates from the ascending and descending tracks, based on corrections using 4 different threshold values in the subwaveform threshold retracker and using the OCOG retracker. The subwaveform threshold retracker with a 0.10 threshold value resulted in the least difference at the two crossovers in central Taiwan and northern China. This result is consistent with the result in Table S3. Note that the difference at the TP-001and TP-062 crossover from OCOG is 0.15 cm yr^-1^, which is the smallest among all differences. However, the VDR from OCOG at this crossover is about -7.50 cm yr^-1^, which is about 3 cm yr^-1^ larger than (in terms of subsidence) the rate determined by precision leveling (the correct rate is about -4.50 cm yr^-1^).

**Table S4:**  Differences between vertical displacement rates from ascending and descending tracks of TP (unit: cm yr^-1^).

| Retracker | TP-051 and TP-164 crossover (Taiwan) | TP-001 and TP-062 crossover (Tianjin, north China) |
| --- | --- | --- |
| No retracking | 3.00 | 11.25 |
| OCOG | 2.43 | 0.15 |
| Subwave 0.1 | 0.20 | 1.58 |
| Subwave 0.2 | 1.14 | 2.81 |
| Subwave 0.3 | 2.03 | 3.21 |
| Subwave 0.5 | 3.30 | 4.81 |

**S4: Time series of height at the TP-EN crossover and at the location of maximum subsidence, near HanfordSan Joaquin Valley, California, USA**

Fig. S6 shows the time series of heights at the crossover of TP-043 and EN-684 (see Fig. 3a) in the SJV. The coordinates of this crossover, located in the Tulare Basin, are 36.1586° N, 119.6594° W. The maximum VDRs from TP and EN along these two passes occur north of the crossover at about 36.25° N and are -6, and -10 cm yr^-1^, respectively. The rates indicate that subsidence here has accelerated from the period of TP (1992–2002) to that of EN (2002–10) and to J2 (2008–15). This acceleration is attributed in part to increased groundwater extraction that accompanies the recent drought in California.

Fig. 5 shows the time series of height at the location of the maximum subsidence (see Fig. 4a) along TP-043. Both Figs. 5 and S6 show trends of decreasing heights with superimposed oscillations in the heights. In Fig. S6, the VDRs from EN and J1 are quite consistent. In the SJV, altimeter-measured heights peaked in summer and were lowest in winter. The oscillations are likely caused by different volume scatters and different soil penetration depths of radar in the dry and wet seasons, and by varying vegetation types that result in variable diffusions of radar pulses.

In Eq. (1), $H_{0}$is the mean height at the mean position (the bin center) for all repeat cycles from a particular satellite mission. The mean position for TP will be different from the mean position for its follow-on mission J1. This is also applicable to the differences between any two missions. At a given bin, the difference between the two mean positions for TP and J1 can vary from few meters to hundreds of meters. As an example, for a gentle slope with a 1% gradient, a 10-m difference in mean position will lead to a 10-cm height difference. Both inter-mission altimeter bias and terrain graident will create an apparent shift between heights from two satellites at a crossover of the two satellites (see Methods). In Figs. 5 and S6, we show both the time series from the original heights and the shifted heights (the shift for a mission is a constant applied to all the heights from this mission).


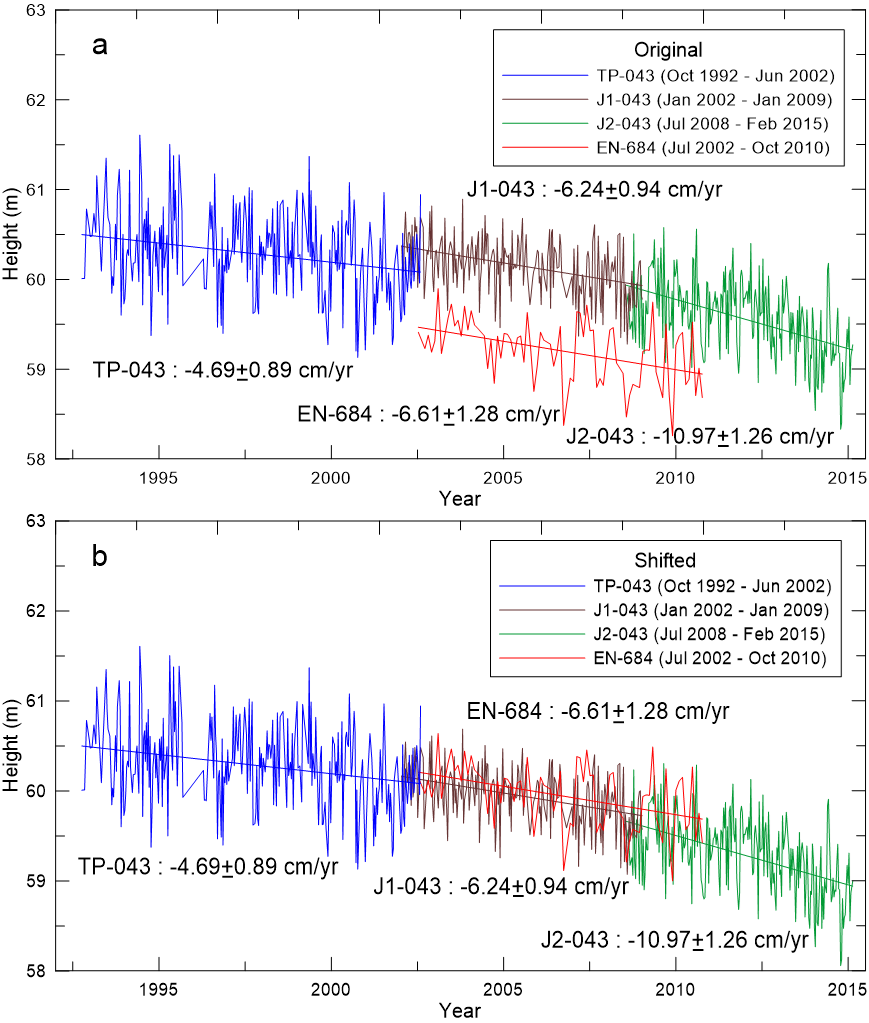


**Fig. S6:** Height changes in San Joaquin Valley, California, from TP, EN, J1 and J2 at the crossover of tracks TP-043 and EN-684 (see Fig. 3a for the location of crossover) from (a) the original heights and (b) shifted heights. The marked differences between EN and J1 heights in (a) are caused partially by different reference ellispoids adopted for the two altimetry GDRs (6378137.0 m versus 6378136.3 m).

**S5: Hanford and Tuku: similarities in distribution of altimeter ground tracks, route of high speed rail and risk of subsidence**

Fig. S7 shows the similarities between Hanford, California, and Tuku, Taiwan, in terms of the distribution of altimeter ground tracks, the route of high speed rail and the risk of subsidence. Both Hanford and Tuku experience the largest subsidence rates (in this study) as seen in the TP-J2 results. The TP-J2 altimeters have provided subsidence rates (Oct 2002–Feb 2015) along the sections of CHSR and THSR near Hanford and Tuku. In Fig. S7b, the red (first priority) - and blue (second priority) -shaded areas within 1.5 km of THSR are areas where groundwater use is restricted. These shaded areas were defined in a project of groundwater restriction use near Tuku that started in 2005 and was completed in 2008 (<http://www.wra.gov.tw/ct.asp?xItem=45677&ctNode=7748&comefrom=lp>, in Chinese). Note that THSR began operation in 2007. As pointed out in the main text, the reduction in the subsidence magnitude and in the subsiding area near Tuku seen in the J2-derived VDRs (Fig. 4b) is due to the measure of groundwater use. THSR in the red-shaded area used to suffer the most severe subsidence problems due to groundwater pumping (Fig. S8) and loading by Expressway No. 78 (Fig. S2). For the planned CHSR, there is a potential operational risk near Hanford due to the large subsidence measured there. However, the restricted groundwater use near Tuku suggests that this measure is effective in reducing the subsidence rate and may be a measure to consider for the CHSR.

With proper data processing, the J2 altimeter and future JASON-3 will continue to provide near real-time monitoring of the severe subsidence in the two areas for safe operations of CHSR and THSR.

**
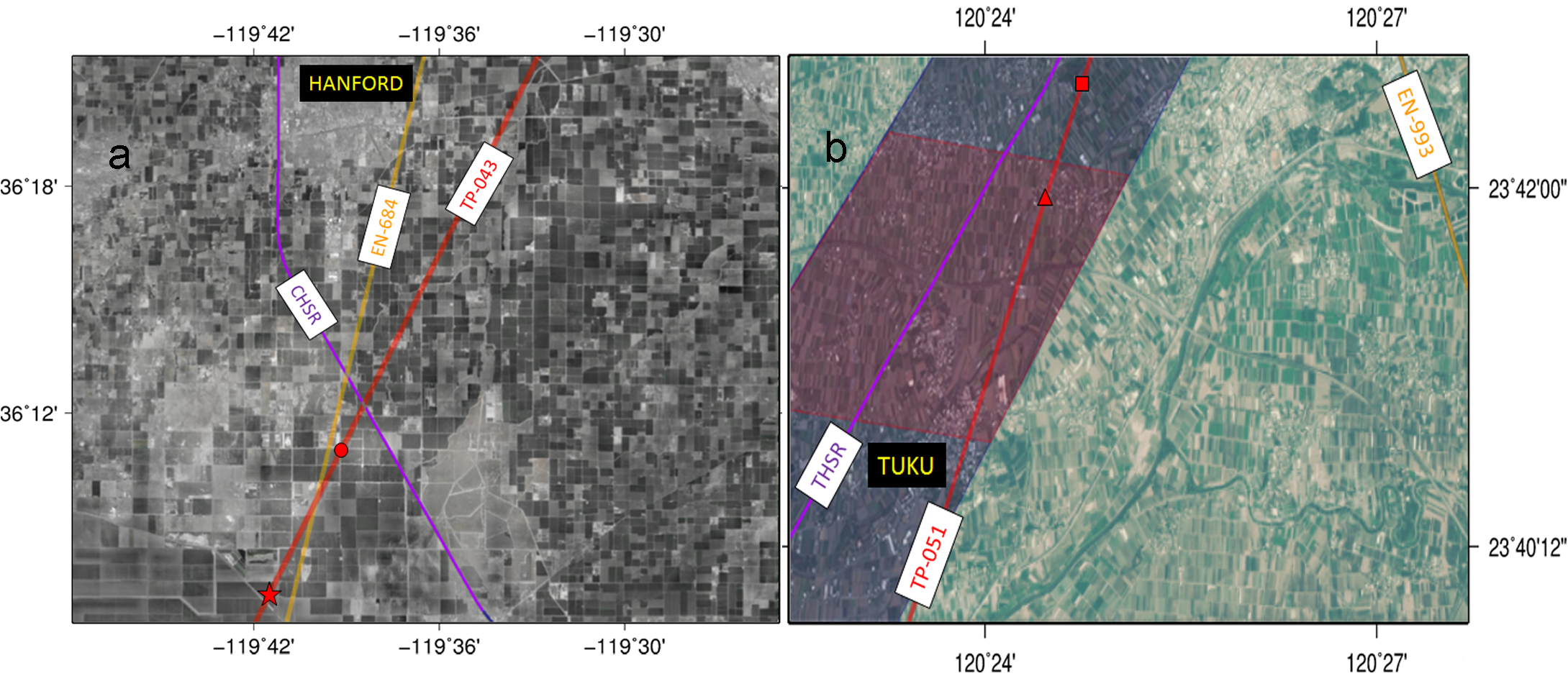
**

**Fig. S7:** (a) CHSR, and ground tracks of TP-043 (same as J1-043 and J2-043) and EN-684 overlapped with an image from the Landsat satellite near Hanford, California. The positions of the red dot and star correspond to the positions of the same symbols shown in Fig. 4a, (b) THSR, and ground tracks of TP-051 and EN-993 overlapped with an image from the SPOT satellite near Tuku Township, Taiwan. The positions of the triangle and square correspond to the positions of the same symbols shown in Fig. 4b. GMT V5.1.3 (<http://gmt.soest.hawaii.edu/)> is used to plot the ground tracks and names. The Landsat image was provided by: U.S. Geological Survey, Earth Resources Observation and Science (EROS) Center, 2015, Landsat products and services: EROS, Glovis Web page, accessed 29 September 2015 at http://glovis.usgs.gov/.


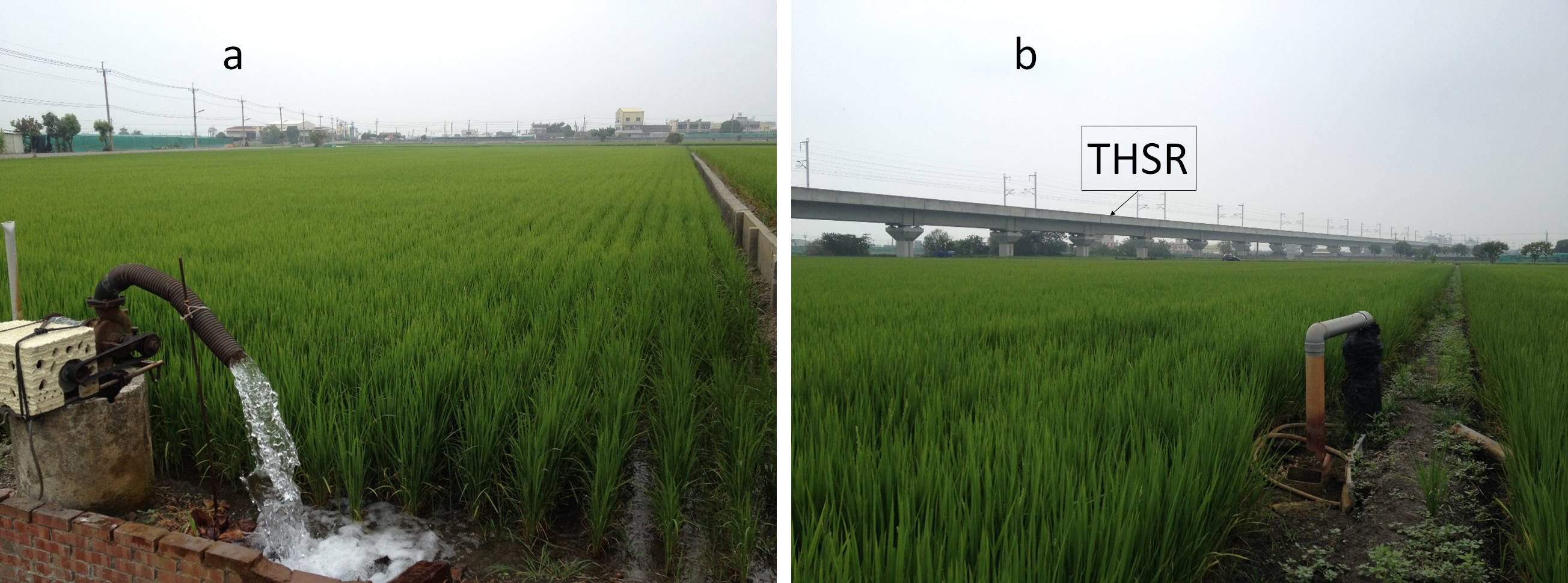


**Fig. S8:** (a) Pumping groundwater to irrigate rice fields near Tuku Township, Taiwan, (b) existing wells are prohibited from pumping groundwater in a restricted area within 1.5 km from the THSR (the shaded-area in Fig. S7b). Irrigation ditches divert water from outside the restricted area. (Photos: R. Kao)

**S6: Vertical displacement rates in the North China Plain from TP, EN,J2, and height time series at crossovers**

Fig. 8 shows the VDRs derived from TP and J2 in the North China Plain. Due to their short repeat period of 10 days, the cross-track spacing (from one ascending track to its neighboring ascending track) is about 314 km. However, the along-track VDRs are useful for detecting cropland subsidence and its time variation. The time series of height changes (original, un-shifted) at the crossovers of TP, EN and J2 at points A-D (Fig. 7) are shown in Figs. S9-12, indicating that the VDRs for different satellite periods can vary substantially. The VDRs in Figs. S9-12 are used to compute the cumulative subsidence values in Fig. 7. During the period when EN and J2 overlap (2008–10), the trends of height changes from the two satellites for this period are very consistent, and the same consistency is also seen in Figs. 5 and S6.

According to Document No. AAA22I-0215-2013-00960 issued by the Water Resource Agency of Tianjin City in 2013 (<http://www.tjzfxxgk.gov.cn/tjep/ConInfoParticular.jsp?id=45399>, in Chinese), pumping of groundwater is prohibited within 1.5 km of the Jin-Qin High Speed Rail (same measure is imposed along THSR near Tuku, Fig. S7). This measure is associated with the deceleration of land subsidence from the EN period to the J2 period seen in Fig. S9. The successful detections of the slowed VDRs due to restricted groundwater use near Tuku (Taiwan) and Tianjin (north China) suggest that satellite altimetry can determine whether a particular management measure to control groundwater use is effective.


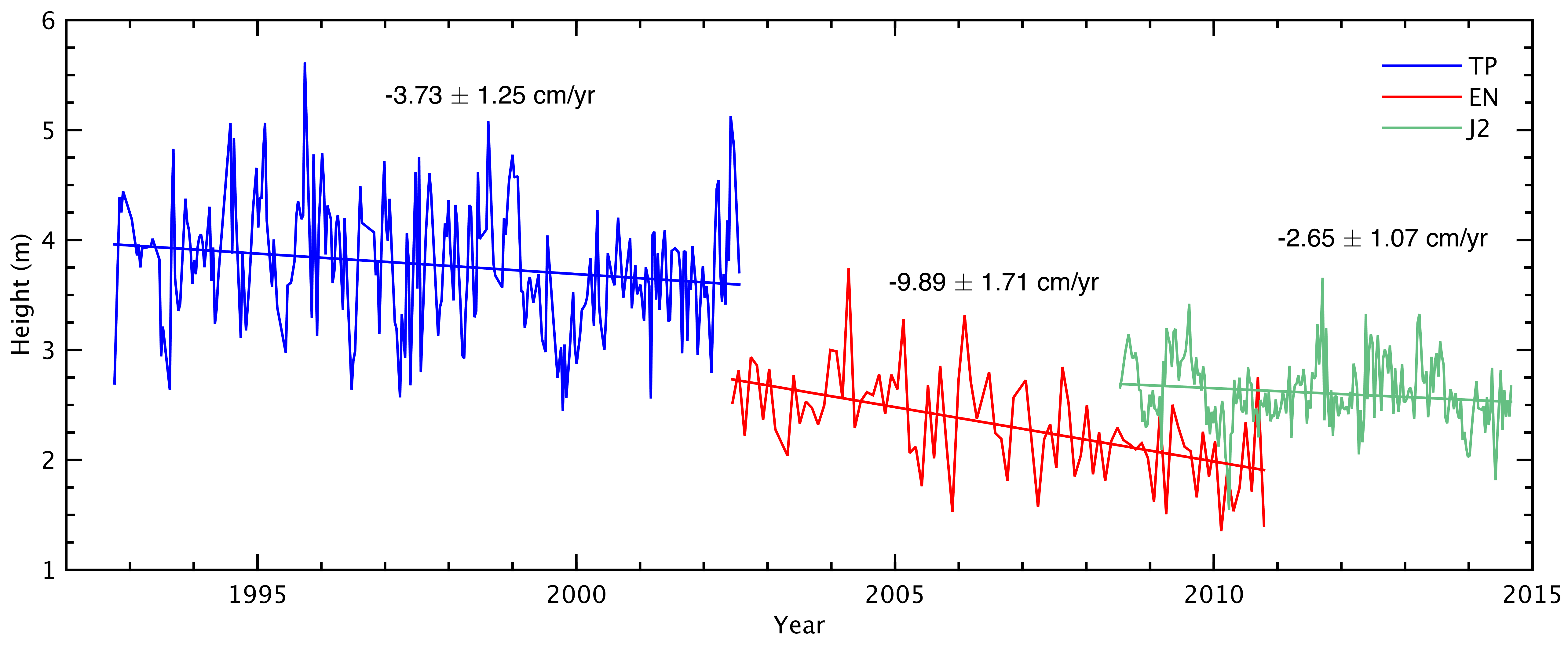


**Fig. S9:** Height time series at a location near Jin–Qin High Speed Rail in the city of Tianjin (A, Fig. 7). This high speed rail was opened in 2013, operating at a maximum speed of 350 km h^-1^.


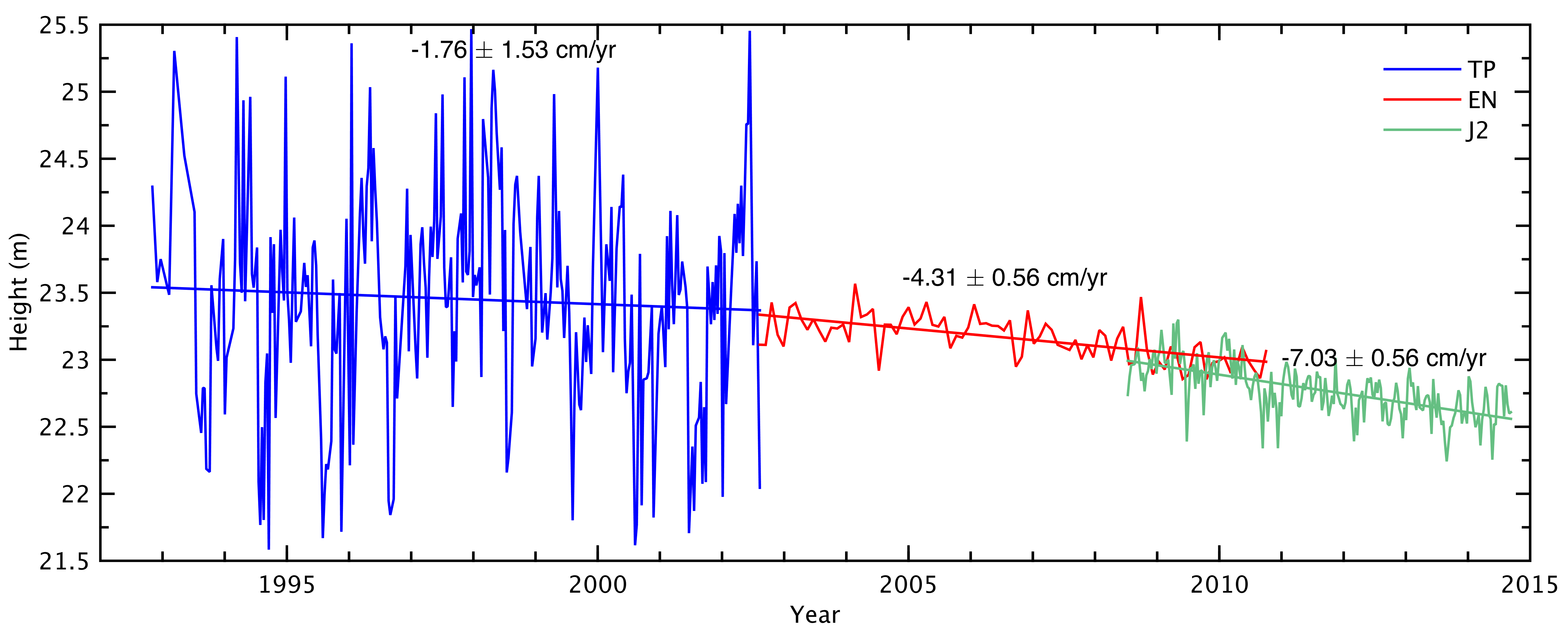


**Fig. S10:** Height time series at a location near Hengshui City, Hebei (B, Fig. 7). Subsidence has accelerated from the time of TP to the time of J2.


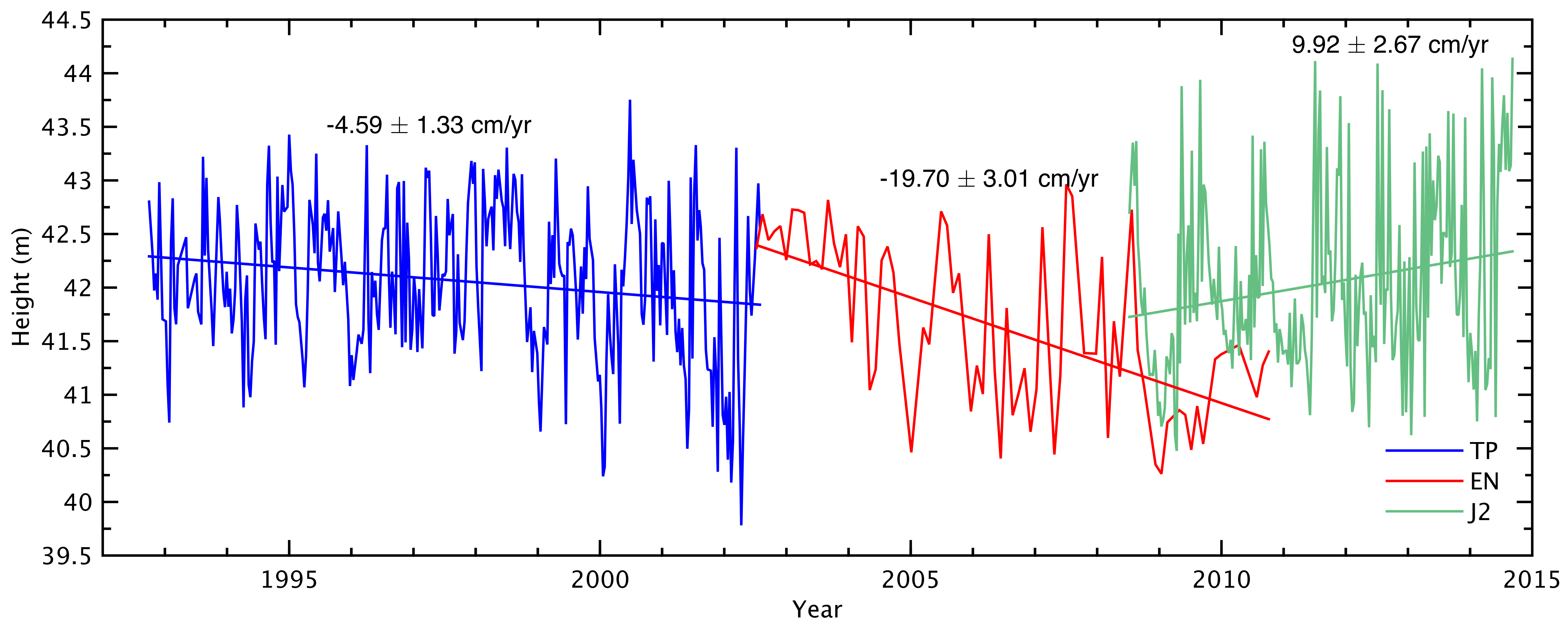


**Fig. S11:** Height time series at a location near Luyi County, Henan province (C, Fig. 7), with J2 heights showing an increasing trend (EN also shows such a trend in the overlapping period 2008–2010).


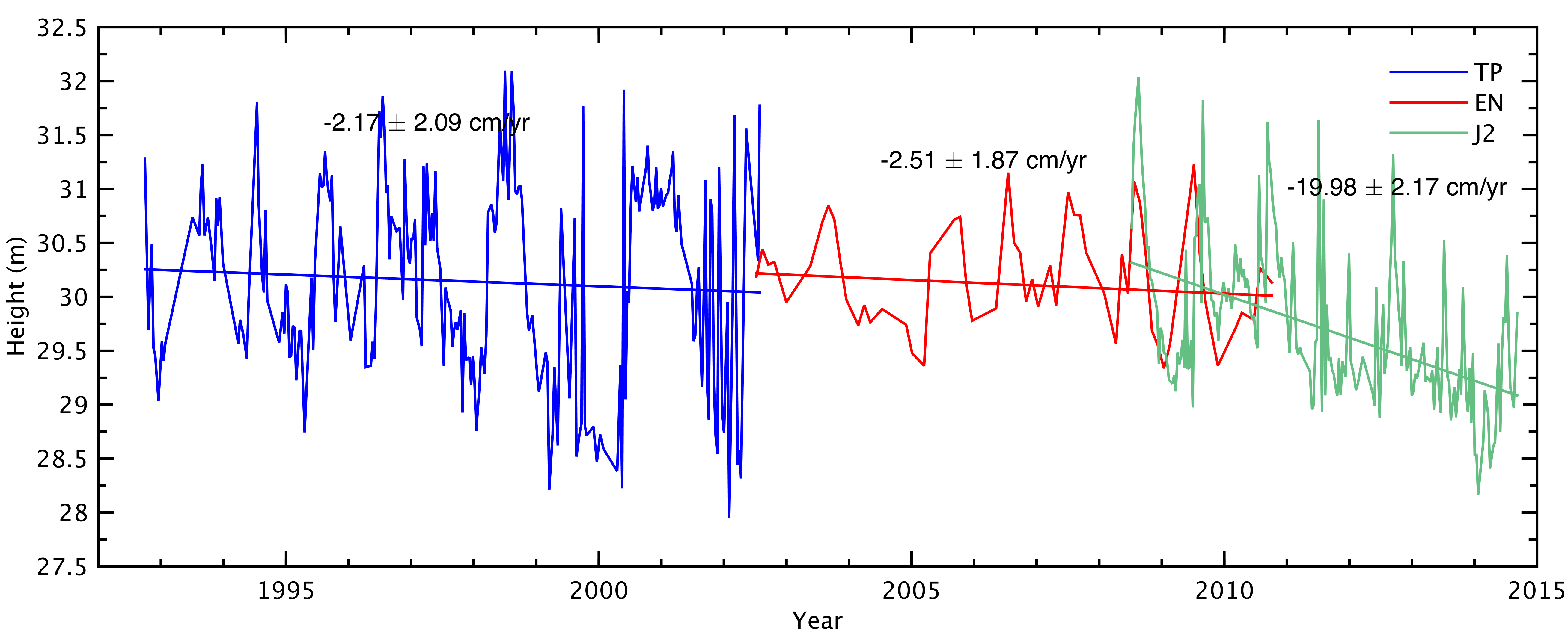


**Fig. S12:** Height time series at a location near Fuyang City, Anhui province (D, Fig. 7), showing a significant accelerating subsidence during 2011–15.

**References for Supplementary Information**

1. Hwang, C., Hung, W. C. & Liu, C. H. Results of geodetic and geotechnical monitoring of subsidence for Taiwan High Speed Rail operation. Natural Hazards **47**, 1-16 (2008).
2. Yang, Y., Hwang, C., Hsu, H., E, D. & Wang, H. A subwaveform threshold retracker for ERS-1 altimetry: A case study in the Antarctic Ocean, Computers & Geosciences **41**, 88-98, doi:10.1016/j.cageo.2011.08.017 (2012).
3. Flament, T. & Rémy, F. Dynamic thinning of Antarctic glaciers from along-track repeat radar altimetry, J. Glaciology **58**, 830-840, doi: 10.3189/2012JoG11J118 (2012).
4. Rémy, F., Flament, T., Michel, A. & Verron, J. Ice sheet survey over Antarctica using satellite altimetry: ERS-2, Envisat, SARAL/AltiKa, the key importance of continuous observations along the same repeat orbit, International Journal of Remote Sensing **35**, 5497-5512, doi: 10.1080/01431161.2014.926419 (2014).
5. Lee, H., Shum, C., Kuo, C., Yi, Y. & Braun, A. Application of TOPEX altimetry for solid earth deformation studies, Terrestrial, Atmospheric and Oceanic Sciences (TAO) **19**, 37-46 (2008).
6. Yang, Y., Hwang, C. & E, D. A fixed full-matrix method for determining ice sheet height change from satellite altimeter: an ENVISAT case study in East Antarctica with backscatter analysis, Journal of Geodesy **88**, 901-914, doi: 10.1007/s00190-014-0730-z (2014).
7. Wingham, D. J., Rapley, C. G. & Griffiths, H. New techniques in satellite tracking system. In: Proceedings of IGARSS’ 88 symposium, Zurich, pp 1339–1344 (1986).
8. Zwally, H. J. & Brenner, A. C. Ice sheet dynamics and mass balance. in L.L. Fu and A. Cazenave (eds.), Satellite Altimetry and Earth Sciences, Academic Press, pp 351-369, doi:10.1016/S0074-6142(01)80154-6 (2001).
